# HSPs drive dichotomous T-cell immune responses via DNA methylome remodelling in antigen presenting cells

**DOI:** 10.1038/ncomms15648

**Published:** 2017-05-31

**Authors:** Lauren B. Kinner-Bibeau, Abigail L. Sedlacek, Michelle N. Messmer, Simon C. Watkins, Robert J. Binder

**Affiliations:** 1 Department of Immunology, University of Pittsburgh, 200 Lothrop Street, Pittsburgh, Pennsylvania 15261, USA; 2 Department of Immunology, Roswell Park Cancer Institute, Elm and Carlton Streets, Buffalo, New York 14263, USA; 3 Department of Cell Biology, University of Pittsburgh, 200 Lothrop Street, Pennsylvania 15261, USA

## Abstract

Immune responses primed by endogenous heat shock proteins, specifically gp96, can be varied, and mechanisms controlling these responses have not been defined. Immunization with low doses of gp96 primes T helper type 1 (Th1) immune responses, whereas high-dose immunization primes responses characterized by regulatory T (Treg) cells and immunosuppression. Here we show gp96 preferentially engages conventional and plasmacytoid dendritic cells (pDCs) under low and high doses, respectively, through CD91. Global DNMT-dependent epigenetic modifications lead to changes in protein expression within these antigen-presenting cells. Specifically, pDCs upregulate neuropilin-1 to enable the long term interactions of pDCs with Treg cells, thereby enhancing suppression of Th1 anti-tumour immunity. Our study defines a CD91-dependent mechanism through which gp96 controls dichotomous immune responses relevant to the therapy of cancer and autoimmunity.

## Abstract (web summary)

Low dose of the heat shock protein gp96 can drive effector T-cell responses, yet high-dose gp96 is immunosuppressive by expanding the regulatory T-cell population. Here the authors explain this dichotomy by showing that high-dose gp96 can drive plasmacytoid dendritic cell expression of neuropilin-1, thus functionally supporting interaction with Treg cells.

The prototypical immunogenic HSP, gp96, is a ubiquitous intracellular chaperone that binds to a variety of endogenous peptides^1^,^2^. After release from damaged cells *in situ* or through administration via vaccination, gp96 engages its receptor CD91 expressed on the surface of antigen-presenting cells (APCs)^3^,^4^,^5^,^6^,^7^,^8^,^9^. CD91 is an endocytic receptor and is responsible for the internalization of gp96-peptide complexes and cross-presentation of the chaperoned peptides^3^,^4^,^5^,^6^,^7^. CD91 also serves as a signalling receptor such that when it is bound by HSPs, intracellular signalling pathways activate nuclear factor (NF)-κB and drive the release of pro-inflammatory cytokines and upregulate co-stimulatory molecules CD86 and CD40 on conventional dendritic cells (cDCs)^8^,^9^. As a result, cDCs stimulated by extracellular gp96 undergo maturation and become highly proficient at priming T helper type 1 (Th1)/CTL (cytotoxic T lymphocyte) responses^5^,^10^. Indeed, vaccination with tumour-derived gp96 primes a potent anti-tumour T-cell response in mice^5^,^10^,^11^ and humans^12^,^13^ and has been used for the clinical immunotherapy of cancer^14^,^15^,^16^. However, priming of Th1 responses is dose-dependent and requires immunization with a microgram (herein called low dose) of gp96. Intriguingly, a tenfold higher dose of gp96 (high dose) primes a suppressive immune phenotype characterized by the preferential expansion of CD4^+^ T regulatory (Treg) cells 10,17,18,19,20,21,22. This response is antigen independent, that is, does not require a specific antigen peptide bound by gp96, and has been used for the prevention of autoimmune responses in diabetes and experimental autoimmune encephalomyelitis mouse models^18^, for the extension of allograft survival in mice^19^ and for suppression of other Th1-mediated immune responses^21^,^22^. The apparent volte-face immune response primed with low-dose versus high-dose gp96 immunization has to date lacked a mechanistic explanation, despite the application of the phenomenon to ameliorate a number of pathological conditions in mice and humans.

For many years, DNA methylation was regarded as a stable and often permanent epigenetic mark that invariably leads to gene silencing. Consequently, its role in controlling transcription and driving immune cellular responses has been neglected. Emerging studies show that in T cells and APCs, active modification of the methylome may occur in response to external stimuli^23^,^24^,^25^,^26^,^27^,^28^,^29^, controlling interleukin-2 production^24^ and proteome modifications in response to pathogens^28^,^29^. We show here that extracellular gp96 differentially engages CD91^+^ APC populations when introduced at low dose versus high dose, driving divergent DNA methylation programs in the respective APCs via activation of DNA methyltransferases (DNMTs). Gp96 can target plasmacytoid DCs (pDCs), upregulating expression of molecules known to support and/or expand a suppressor immune phenotype. We show that in gp96-stimulated pDCs, DNA methylation changes result in upregulation of neuropilin-1 (Nrp1) expression, leading to stabilization of pDC-Treg cell interactions. Accordingly, depletion of pDCs eliminates high-dose gp96-mediated suppression *in vivo* and results in maintenance of CTL responses. Hence, at a cellular and molecular level, exogenous gp96 at high dose instigates the development of regulatory Nrp1^+^ pDCs that enforce Treg-mediated tolerance.

## Results

### CD91^+^ DCs are required for gp96-mediated suppression

**Figure 1:**
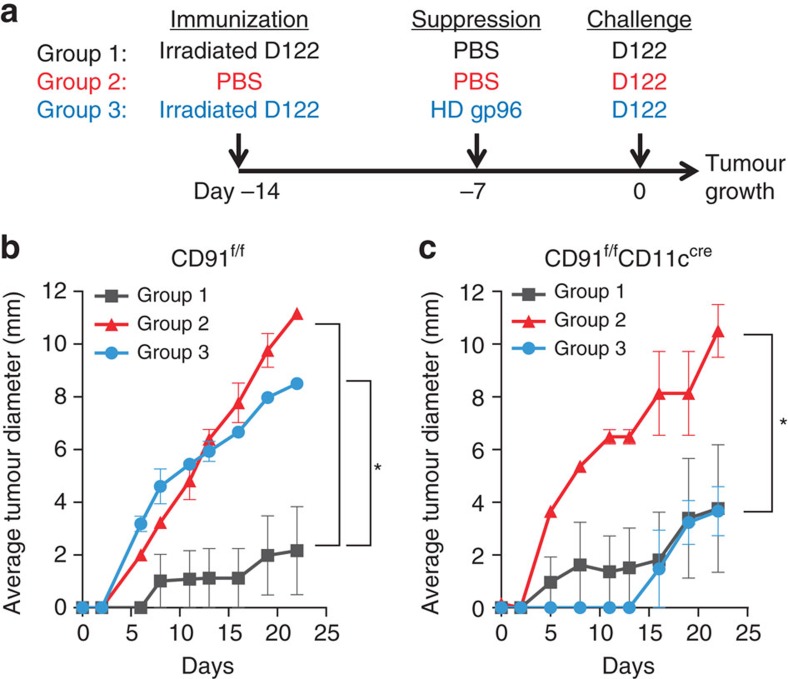
High-dose gp96-mediated immune-regulatory response requires CD91^+^ DCs. (**a**) The experimental set-up to test the role of CD91^+^ DCs in HSP-mediated immune-regulatory response. Immunization was carried out using irradiated D122 (Groups 1 and 3) or control PBS (Group 2). Suppression of anti-tumour immunity, primed by irradiated D122, was mediated by high-dose (HD) gp96 treatment (Group 3) or control PBS (Groups 1 and 2). All mice were challenged with D122 and tumour growth was monitored. (**b**) Tumour growth in wild-type littermates (CD91^f/f^). (**c**) Tumour growth in mice lacking expression of CD91 in CD11c^+^ cells (CD91^f/f^CD11^cre^). Shown is the average tumour diameter ± s.e.m. *n*=3–5/group, data are from one representative experiment of three independent experiments. (The value of s.e.m. in Group 3 is too small to be seen in the graph). Data are represented as mean±s.e.m. *ns* not significant, *P<0.05 versus control (area under curve).

CD91 is an endocytic and signalling receptor for gp96, and its selective deletion in cDCs renders mice incapable of priming Th1/CTL immune responses against tumours when immunized with low-dose gp96 (ref. 30). We tested whether CD91 was required to prime immune suppression in a murine model of cancer when mice were immunized with high-dose gp96. Towards this goal, we have generated mice that are selectively deficient in CD91 expression on CD11c^+^ cells (CD91^f/f^CD11c^cre^) and characterized their phenotype^30^. These mice have normal numbers of APCs (including cDCs and pDCs), T cells, and B cells at steady state^30^ and were used in a gp96-mediated suppression assay (Fig. 1a). CD91^f/f^CD11c^cre^ or wild type littermates (CD91^f/f^) were immunized with irradiated tumour cells. Mice were treated with high-dose gp96 followed by tumour challenge. Tumour growth was monitored in all mice by measurement of tumour in two perpendicular axes. Regardless of CD91 expression, mice immunized with irradiated tumour cells only (Group 1) were able to reject a subsequent challenge with that tumour (Fig. 1b,c), while unimmunized mice developed progressive tumours (Group 2). In a separate cohort (Group 3), immunized mice were administered high-dose gp96 7 days before tumour challenge. Vaccinated CD91^f/f^ mice were unable to limit tumour growth following high-dose gp96 administration (Fig. 1b). However, similarly treated CD91^f/f^CD11c^cre^ mice remain protected against challenge (Fig. 1c). These results indicate that CD91^+^CD11c^+^ APCs were necessary for high-dose gp96-mediated suppression of protective anti-tumour immunity in vaccinated hosts. This observation was also dependent upon the dose of gp96 since mice treated with low-dose gp96 failed to suppress anti-tumour immunity (Supplementary Fig. 1), consistent with previous reports^17^.

### Dose-dependent interactions of gp96 with DC populations

**Figure 2:**
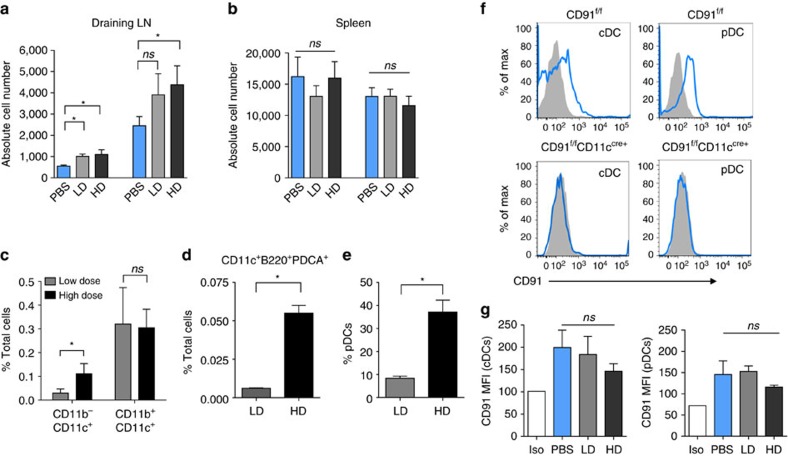
Preferential targeting of CD11c^+^ cells at high-dose gp96. (**a**,**b**) Mice were immunized intradermally with low-dose (LD) or high-dose (HD) gp96 or given PBS. After 18 h, draining lymph node (**a**) and spleens (**b**) were harvested and absolute numbers of CD11b^+^ and CD11c^+^ populations were analysed. *n*=5/group, data are from one representative experiment of three independent experiments performed. (**c**–**e**) Mice were immunized with low-dose (LD) or high-dose (HD) gp96_A488_. Lymph nodes were harvested 8 h later. Lymph node cells were analysed for A488 positivity by flow cytometry using CD11b and CD11c cell surface markers. Each population is shown as a percentage of the total lymph node cells (**c**). (**d**) The percent of pDCs in the CD11b^−^CD11c^+^ population from (**c**) that are A488^+^ is shown as a fraction of total lymph node cells. (**e**) The percent of pDCs that are A488^+^ is shown. Data are from one representative experiment of three independent experiments. (**f**) Histograms depict CD91 staining within representative lymph node cDC (CD11b^+^CD11c^+^) or pDC (CD11c^low/+^CD11b^−^ B220^+^PDCA1^+^) populations from naive CD91^f/f^ or CD91^f/f^CD11c^cre^ mice. (**g**) Mice were immunized with low-dose (LD) or high-dose (HD) gp96 or given PBS. At 18 h post-immunization, lymph nodes were harvested and stained for CD91 in pDCs and cDCs. Shown are CD91 MFI values for each population. *n*=5/group, data are from one representative experiment of three independent experiments. Data are represented as mean±s.d. *ns*, not significant, *P<0.05 (Student's *t*-test used in **c**,**d**,**e**; one-way ANOVA used in **a**,**b**,**g**).

We have previously shown that immunization with low-dose gp96 leads to priming of anti-tumour immune responses^1^,^2^,^10^,^11^,^12^,^30^. This is in stark contrast to responses primed by high-dose gp96 shown here. To understand this operational dichotomy in greater detail, we investigated the CD91^+^CD11c^+^ cells necessary for gp96-mediated immune responses. We first analysed the two major APC types within this population, both of which express high levels of CD91 (ref. 31). Mice were immunized with low-dose or high-dose gp96 or administered PBS. Lymph nodes or spleens were isolated after 18 h and cells were analysed by flow cytometry. The total number of CD11c^+^CD11b^+^ cells and CD11c^+^CD11b^−^ cells in the lymph nodes of immunized mice were increased 2–3-fold over samples from unimmunized mice regardless of whether mice were immunized with low-dose or high-dose gp96 (Fig. 2a). Such increases were not observed in spleen samples (Fig. 2b). Thus, comparative differences in the number of cells in these two populations in lymph nodes by themselves could not account for altered immune responses observed after immunization with low-dose versus high-dose gp96. We next examined the APCs that engage gp96 following immunization at either dose. Gp96 was labelled with Alexafluor 488 (A488) and low-dose or high-dose gp96_A488_ was administered to mice. Draining lymph nodes were examined by flow cytometry after 8 h and analysed for A488 fluorescence. CD11c^+^CD11b^+^ cells were preferentially engaged by gp96 and this appeared unrelated to the dose of gp96 used for immunization (Fig. 2c). At low-dose gp96, CD11c^+^CD11b^+^ cells (cDCs) cross-present HSP-chaperoned peptides, release pro-inflammatory cytokines that promote Th1 immunity and are responsible for the induction of anti-tumour immunity^31^. However, CD11c^+^CD11b^−^ cells endocytosed ∼2.6-fold more gp96 at high dose versus low dose (Fig. 2c). We thus focused on CD11c^+^CD11b^−^ DC for two reasons; first, this cell population incorporated more gp96 at high dose versus low dose, and second, the CD11c^+^CD11b^−^ population includes pDCs, known for their immunosuppressive properties^32^,^33^. Of the total number of cells in the lymph node, 0.055% were gp96^+^ pDCs (CD11c^low/+^CD11b^−^B220^+^PDCA^+^) when mice were administered high-dose gp96_A488_, significantly higher that 0.005% with low-dose gp96_A488_ immunization (Fig. 2d). Upon further analysis, the number of pDCs that were gp96_A488_^+^ increased fivefold between low dose and high dose (Fig. 2e). Importantly, we examined pDCs in CD91^f/f^CD11c^cre^ mice for CD91 expression given their low CD11c expression. Lymph nodes were first isolated from CD91^fl/fl^CD11c^cre^ or CD91^fl/fl^ mice and analysed for cDCs and pDCs. We show for the first time that CD91 expression was undetectable on pDCs isolated from CD91^fl/fl^CD11c^cre^ mice and its deletion was as complete as that for cDCs (Fig. 2f). Interaction of gp96 with different populations of DCs is strictly correlated with^31^,^32^, and dependent on^3^,^4^,^5^,^30^, the levels of CD91 that they express. This correlation is also true when the gp96-mediated phenotype is measured in DCs. pDCs expressed CD91 to similar levels seen in cDCs (Fig. 2f). Importantly the levels of CD91 did not change following immunization of mice with any dose of gp96 (Fig. 2g).

### DNA methylome remodelling occurs in response to gp96

**Figure 3:**
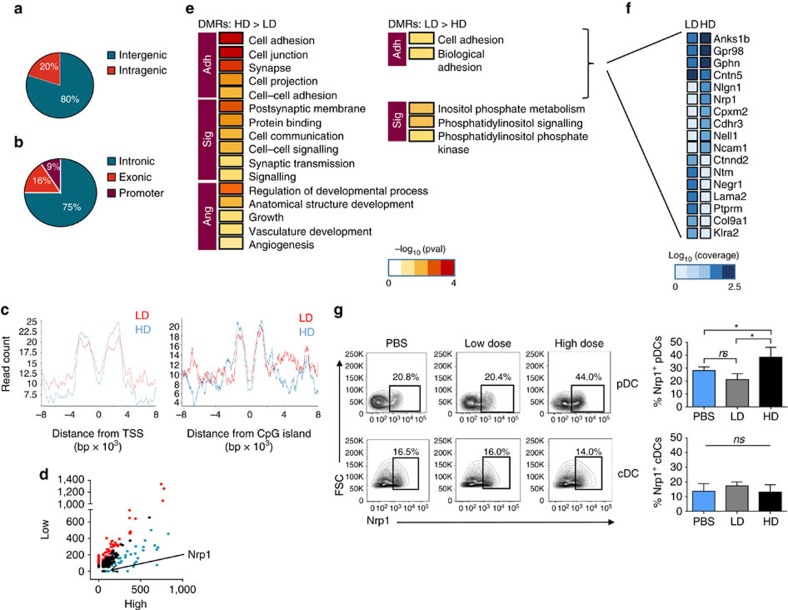
Differential regulation of adhesion molecules via DNA methylation in response to gp96. Mice were immunized with low-dose (LD) or high-dose (HD) gp96 or treated with PBS. DNA was purified from isolated CD91^+^ lymph node cells, fragmented and methylated sequences were enriched using methyl-binding domain protein (MBD). Methylated DNA was sequenced. (**a**) Pie chart shows distribution of total DMRs on intergenic versus intragenic regions. Here intergenic is defined as any sequence >2,000 bp away from any annotated gene, and intragenic is defined as any sequence within 2,000 bp of an annotated gene. (**b**) Intragenic DMRs were analysed for distribution on gene elements (promoters, introns and exons). (**c**) Average signal profile for methylation enrichment across transcription start sites (TSS) and CpG islands. (**d**) Scatter plot of coverage scores for all methylated genes in low-dose (LD) and high-dose (HD) samples. Any genes defined as DMRs are highlighted. (**e**) Gene Ontology analysis of all DMRs identified in low dose (LD) and high dose (HD). Adh=adhesion; Sig=signalling; Ang=angiogenesis. (**f**) Sample genes within the adhesion gene family set, with log(coverage) shown. (**g**) Mice were immunized with low-dose (LD) or high-dose (HD) gp96 and draining lymph nodes were harvested and stained with markers for pDC or cDC plus Nrp1. Cells were analysed by flow cytometry for changes in percentage of Nrp1^+^ in respective pDC and cDC gates. One set of representative flow plots is shown. *n*=5/group, data are from one representative experiment of three independent experiments. Bar graphs on the right are the average percentages from multiple experiments. Data are represented as mean±s.d. *ns*, not significant, *P<0.05 (one-way ANOVA).

DNA methylation is a critical modification responsible for transcriptional regulation of cytokines, growth factors, molecules involved in synapse formation and other aspects of immunologic responses^23^,^24^,^25^,^26^,^27^,^28^,^29^. Activation of NF-κB can lead to recruitment of DNMTs to specific target genes^34^,^35^. Given that NF-κB is a critical downstream factor of CD91-HSP signalling in DCs^8^,^9^,^32^, we hypothesized that DCs undergo methylome remodelling in response to gp96 immunization. We examined DNA methylation first in total CD91^+^ cells following immunization of mice with low-dose or high-dose gp96 to determine if changes in methylation was a mechanism for controlling the divergent T-cell immune response. A whole-genome methyl-seq approach was taken using MBD-based purification of methylated DNA. Mice were immunized with low-dose or high-dose gp96 or given PBS. Eighteen hours post-immunization, mice were killed and CD91^+^ cells were isolated by fluorescence-activated cell sorting (FACS) from draining lymph nodes. The target cell population and time point were chosen in accordance with the outcomes in Fig. 2. We generated an average of 45 million filtered and aligned single-end reads per sample (Supplementary Table 1). Methylated DNA was sequenced to ∼11-fold coverage and analysed for differential methylation. Here intergenic regions are defined as any sequence >2,000 bp distal from annotated genes including alternate promoter and cis-acting regulatory sequences. Intragenic regions include sequences <2,000 bp up or downstream from an annotated gene or within the gene body. Consistent with previous reports^29^, the majority of detected differentially methylated regions (DMRs) which occurred in intragenic regions were located within introns, with a small percentage occurring at promoter regions (Fig. 3a,b). Methylation was largely not present at transcription start sites (TSS) and CpG islands (Fig. 3c), again consistent with what others have reported for DNA methylation sequencing^29^. From this data set, 184 intergenic and 55 intragenic DMRs were identified exhibiting variance between low-dose and high-dose gp96 immunized cohorts (Supplementary Table 2). Of the 55 intragenic DMRs, 29 were hypermethylated in high dose and 26 were hypermethylated in low dose. Methylation scores for all detected peaks were plotted, with DMRs highlighted to show divergence between samples (Fig. 3d). Using parameters described in Methods, gene ontology analysis was performed on intragenic DMR genes and showed that the bulk of intragenic DMRs occur within pathways, which primarily regulate cell–cell contact and adhesion, intracellular signalling and angiogenesis (Fig. 3e; Supplementary Table 3). A representative list of genes, most of which are expressed by APCs (Supplementary Table 3), enriched for cell–cell interaction are shown (Fig. 3f). We then focused on differential methylation and expression of target adhesion molecules in CD91-expressing APCs since APC-T cell contact is critical for proper T-cell activation. One of the identified adhesion molecules, Nrp1, known to control Treg responses when expressed in pDCs^36^,^37^, was notable. Nrp1 has the most well-established role in controlling DC-Treg interaction at steady state^36^,^37^ and regulating Treg activation in the tumour microenvironment^38^. *Nrp1* showed intragenic methylation in samples from high-dose gp96 immunized mice but not from low-dose immunized mice (Fig. 3f). To determine the effect of gene methylation on Nrp1 protein expression, mice were immunized with gp96 and 18 h later draining lymph nodes were stained for markers of cDCs and pDCs and analysed for Nrp1 expression by flow cytometry. The percentage of Nrp1^+^ pDCs was significantly increased when mice were immunized with high dose but not low-dose gp96 (Fig. 3g). Interestingly, cDCs showed no increase in Nrp1 expression following gp96 immunization at either dose (Fig. 3g). Although cDCs did not alter expression of Nrp1 following gp96 treatment, they responded to gp96 in other ways including upregulation of CD86 and major histocompatibility complex II (Supplementary Fig. 2) consistent with our previous studies^8^. No changes in Nrp1 expression were detected in B cells, T cells, NK cells and other CD11c^+^ DCs (Supplementary Fig. 3) at any dose of gp96 tested.

### *Nrp1* methylation occurs in pDCs in response to gp96

**Figure 4:**
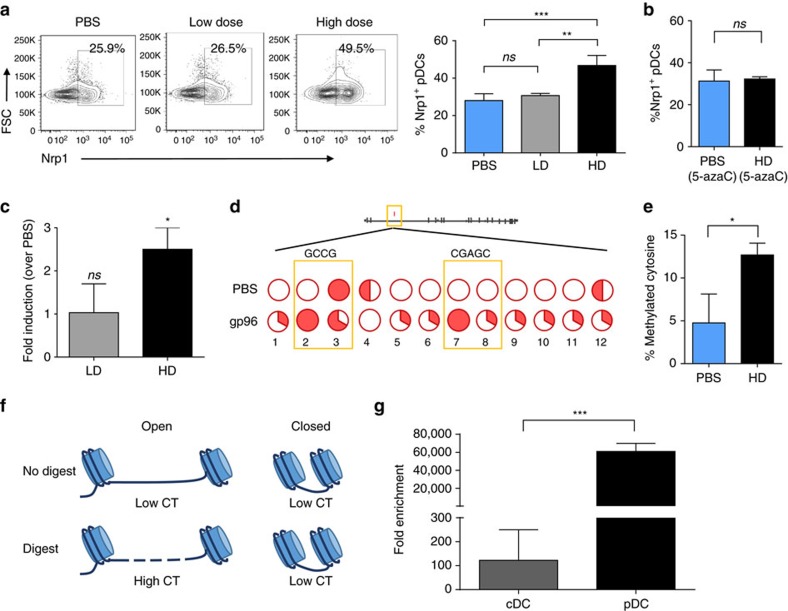
pDCs increase *Nrp1* intronic methylation and protein expression in response to gp96 stimulation. (**a**) Splenic pDCs were isolated, treated with low-dose (LD) or high-dose (HD) gp96 *ex vivo*, and Nrp1 expression was measured by flow cytometry. (**b**) Splenic pDCs were isolated from 5-azaC treated mice and stimulated with HD gp96 *ex vivo* for 18 h as in **a**. pDCs were stained with for pDC markers and Nrp1, and analysed by flow cytometry. Percent of Nrp1^+^ pDCs of total pDCs is shown. (**c**) Splenic pDCs were isolated, treated with low-dose (LD) or high-dose (HD) gp96 *ex vivo*, and Nrp1 expression was measured by qPCR. In a (bar graph), **b**,**c**, data are pooled from three independent experiments. (**d**) Schematic of methylation within *Nrp1* at intron 2 from whole-genome methyl seq data. Splenic pDCs were isolated from naive mice and stimulated with high-dose (HD) gp96. Purified DNA was analysed by clonal bisulfite sequencing. Each line of bisulfite seq data represents one treatment group. Filled slices of the pie-charts signify methylated samples; open slices signify unmethylated samples. Only differentially methylated cytosines are shown; both CpG and non-CpG are included. (**e**) Percent of methylated cytosines of total cytosines as measured by clonal bisulfite sequences is shown. Data are from one representative experiment of two independent experiments. (**f**) Schematic of chromatin accessibility experiment. (**g**) Chromatin was purified splenic pDCs or cDCs isolated from naive mice and digested. Chromatin accessibility at the Nrp1 region of interest was assessed by qPCR. Fold enrichment was calculated using the formula: FE=2̂(NseCT-noNseCT) × 100%. *n*=3/group, data are pooled from three independent experiments. Data are represented as mean±s.d. *ns* not significant, *P<0.05, **P<0.01, ***P<0.001 (Student's *t*-test used in panels **b**,**c**,**e**,**g**; one-way ANOVA used in **a**).

We established an *in vitro* system to examine the specific effect(s) of gp96 on pDCs observed in high-dose immunized mice. Based on our estimates of internal volumes and number of cells exposed to injected gp96, we used 200 and 10 μg ml^−1^ gp96 in culture to represent high-dose and low-dose gp96 *in vivo*, respectively. Nrp1 expression on the surface of pDCs was examined by flow cytometry following treatment of cells in culture with high dose or low dose. As shown in Fig. 4a, the percent of Nrp1^+^ pDCs is enhanced when cells were treated with high-dose gp96 but not low-dose gp96 or PBS, recapitulating the effects seen *in vivo* (Fig. 3g). We next verified that DNA methylation was responsible for increased Nrp1 expression by inhibiting DNA methylation. Mice were administered 5-azacytidine (5-azaC), a potent inhibitor of DNMTs, 72 h before isolation of pDCs. pDCs from 5-azaC treated or untreated mice were incubated *in vitro* with high-dose gp96 and analysed for Nrp1 expression by flow cytometry. 5azaC completely blocked the upregulation of Nrp1 in pDCs treated with gp96 *in vitro* (Fig. 4b). We measured *Nrp1* messenger RNA by quantitative PCR (qPCR) in pDCs treated with low-dose or high-dose gp96. Consistent with protein expression, *Nrp1* messenger RNA increased significantly when cells were treated with high-dose gp96 but not low-dose gp96 (Fig. 4c). Following the consistent Nrp1 protein expression patterns in pDCs *in vivo* and *in vitro* we next confirmed the methyl-seq data in pDCs stimulated with high-dose gp96 *in vitro.* pDCs were cultured in the presence of high-dose gp96, and analysed at a single base resolution of the *Nrp1* intronic DMR by clonal bisulfite sequencing (Fig. 4d). Bisulfite-treated DNA was PCR amplified with specific primers. PCR amplicons were cloned and sequenced and are indicated as circles, which represent individual cytosines differentially methylated in either sample (Fig. 4d). Consistent with the methyl-seq data set (Fig. 3), the percentage of total methylated cytosines within intron 2 of *Nrp1* was significantly increased in gp96-treated pDCs versus untreated pDCs (Fig. 4e). These data are also consistent with previous observations, in that methylation within such non-promoter regions was associated with enhanced protein expression^39^. The effect of DNA methylation initiated at the *Nrp1* locus on protein expression is reflected in the increase in protein expression (Fig. 4a). To understand the disparity in methylation patterns at the *Nrp1* locus and Nrp1 expression in pDCs and cDCs, we examined chromatin accessibility in naive cells (Fig. 4f). Chromatin was extracted from cDCs or pDCs and digested with nuclease. The *Nrp1* locus was then amplified and quantified with specific primers by qPCR. In this chromatin accessibility assay, when the DNA is in a heterochromatin (closed) state, it is inaccessible to nuclease digestion and will result in insignificant CT shifts between digested and undigested samples (Fig. 4f). DNA in a euchromatin (open) state will be susceptible to nuclease digestion and result in large CT shifts upon amplification. We observed that at a basal state, *Nrp1* in pDCs is in an open confirmation and thus more accessible, as revealed by the large fold enrichment score (Fig. 4g). In contrast, *Nrp1* was in a closed chromatin state in cDCs. These data indicate that pDCs are more poised for regulation of *Nrp1* at a genetic level.

### Gp96 activates DNMT1 punctae formation in cDCs and pDCs

**Figure 5:**
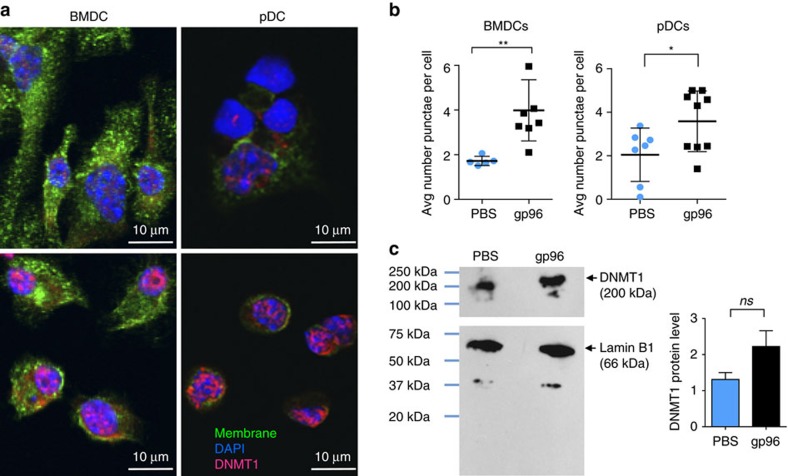
DNMT1 forms punctae in DC nuclei following gp96 stimulation. (**a**) Indicated DCs were stimulated with gp96 or treated with PBS *in vitro* on coverslips for 6 h. Cells were stained and analysed by confocal microscopy. (**b**) DNMT1 punctae were quantified using NIS Elements software and in-house algorithms as described in Methods. Each point represents the average punctae per cell from one field of view. Data are from one representative experiment of three independent experiments. (**c**) Nuclear extracts from cDCs after 18 h gp96 stimulation were harvested for western blot analysis. Following transfer of nuclear extract protein from the gel to the western blot membrane, the membrane was cut in half horizontally. The top half was probed for DNMT1 and the bottom half was probed for Lamin B1 as a loading control. Data are from one representative experiment of three independent experiments. Data are represented as mean±s.d. *ns*, not significant, *P<0.05, **P<0.01 (Student's *t*-test).

DNA methylation is catalysed by DNMT1, DNMT3a and DNMT3b. To confirm that methylome remodelling is associated with increased enzyme activity, we tested whether cDCs and pDCs cultured in the presence of gp96 modify nuclear organization of DNMTs. We also included a range of other (conventional) dendritic cells in this experiment including bone marrow-derived dendritic cells (BMDCs), peritoneal exudate cells (PECs) and splenic DCs. DCs were separately cultured in the presence of gp96 for 6 h on glass coverslips. The cells were then probed by immunocytochemistry for formation of punctate DNMT structures within the nucleus, an established indicator of binding of DNMT to target genes^40^. Using the analysis tools described in Methods, the average number of DNMT punctae per cell was calculated. We observed a significant and similar increase in DNMT1 punctae in gp96-stimulated pDCs and all cDCs after 6 h (Fig. 5a,b, Supplementary Fig. 4a,b) compared with the diffuse with little to no punctae in PBS-treated cells. We did not observe punctae formation with DNMT3a in either cell type following gp96 treatment. Gp96-mediated DNMT1 punctate enrichment was accompanied by elevated, but not significantly increased, DNMT1 protein amount in nuclear extracts purified from cDCs (Fig. 5c). Therefore, changes to DNMT1 nuclear architecture, rather than overall increased DNMT1 expression, were initiated by gp96 in responding cells. DNMT1 punctae formation was not a result of proliferation as treated cells did not significantly proliferate throughout the duration of the experiment and lacked co-localization of DNMT with Ki67 (Supplementary Fig. 4c) which is expressed at the S-phase during cell proliferation and DNA replication^41^. DNMT1 punctae was dependent on NF-κB activation as shown by its abrogation when cells were treated priorly with the NF-κB inhibitor cardamonin (Supplementary Fig. 4b).

### pDCs stabilize interactions with Treg in response to gp96

**Figure 6:**
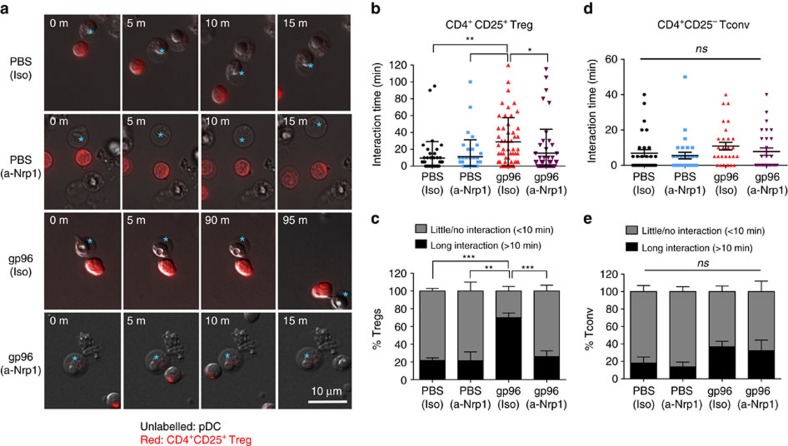
gp96-stimulated pDCs increase interaction with Treg in a Nrp1-dependent manner. (**a**–**e**) Splenic pDCs were plated on coverslips with or without high-dose gp96, and in the presence or absence of Nrp1 blocking antibody (α-Nrp1) or isotype control antibody (Iso). Before imaging, Treg (CD4^+^CD25^+)^ or Tconv (CD4^+^CD25^−^) were labelled with Cell Tracker Red dye and added to the pDC culture dishes at a 2:1 DC:T-cell ratio. Images were acquired at 5 min intervals for a total of 1–2 h. Interaction and motility were analysed. (**b**) Treg were tracked using NIS Elements tracking software. Representative Treg-pDC interactions are shown for each treatment group for the indicated times. The interaction times of Tregs with pDCs from multiple images are quantified. All Tregs (including those that failed to interact with pDC) are included. (**c**) The average percentage of Tregs which undergo long (>10 min) or short to no (0–10 min) interactions with pDCs were calculated for each group. (**d**,**e**) Tconv were analysed similarly to Treg. Total interaction time (**d**) and percentage of long interaction (**e**) are shown. All data are from one representative experiment of 2–3 independent experiments. Data are represented as mean±s.d. *ns*, not significant, *P<0.05, **P<0.01 (one-way ANOVA).

Nrp1 expression by pDCs facilitates long-term homotypic Nrp1-Nrp1 (refs 36, 37) or heterotypic Nrp1-Sema4a (ref. 38) interaction with Treg cells. Having established that both high-dose gp96 immunization and *in vitro* gp96 stimulation result increased Nrp1^+^ pDCs, we investigated whether *in vitro* gp96-stimulated pDCs prolonged interaction with Tregs as measured by time-lapse live cell microscopy. pDCs and Tregs were isolated from spleens. pDCs were cultured for 18 h with high-dose gp96, and for the last 2 h in the presence or absence of Nrp1 blocking or isotype control antibody. CellTracker Red-labelled CD4^+^CD25^+^ Tregs were added to the culture and imaged for 2 h, with frames taken at 5 min intervals. Figure 6a shows representative pDC-Treg interactions over time. High-dose gp96 significantly increased interaction time between pDCs and Tregs and this interaction could be blocked by Nrp1 antibodies but not by an isotype control antibody (Fig. 6a–c). Interactions were considered ‘long' if the pDC-Treg interaction lasted >10 min. Any interaction that lasted <10 min was deemed transient, and placed in the ‘little/no interaction' category. In all samples, some baseline level of long interactions were observed, which is in agreement with Tregs scanning for antigen^42^ and/or recognition of self-antigen to maintain the Treg lineage^43^. However, Nrp1 antibody did not have an overall inhibitory effect on baseline long interactions. Over the course of the experiment the gp96-treated pDCs did not undergo maturation and maintained an immature phenotype, further supporting previous findings that Nrp1-expressing immature DCs efficiently interact with Treg (Supplementary Fig. 2)^37^. Furthermore, we tested whether this phenomenon is Treg-specific, or if gp96-stimulated pDCs increase interaction with conventional T cells (Tconv). CD4^+^CD25^−^ Tconv were co-cultured with pDCs treated in the same way as for the Treg in Fig. 6a–c. There was no change in pDC-Tconv interaction regardless of gp96 or antibody treatment (Fig. 4d,e), suggesting that this is a Treg-specific event and further supports the dependence of Nrp1 since Tconv do not express high levels of Nrp1. Although low dose treatment of pDCs led to a marginal increase in pDC-Treg stabilization it was significantly lower than stabilization achieved with high dose (Supplementary Fig. 5). Stimulation of pDCs by gp96, therefore, prepares these pDCs for longer interaction with Treg by increasing expression of Nrp1.

### pDCs are required for gp96-mediated immune suppression

**Figure 7:**
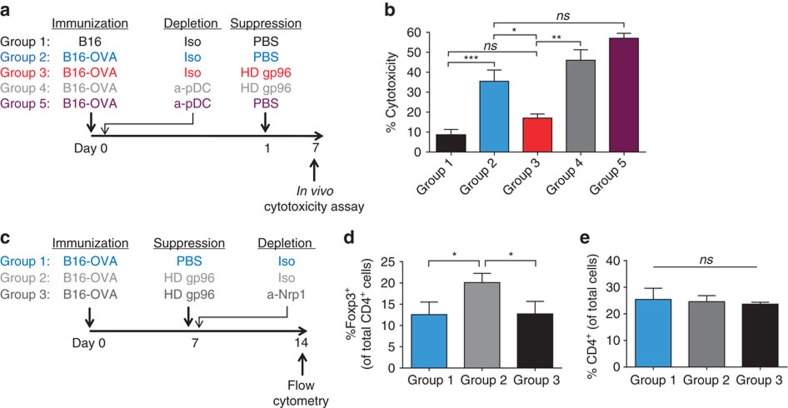
pDCs are required for efficient Nrp1-dependent suppression of CTL-mediated cytotoxicity by high-dose gp96. (**a**) Schema for experiment. Mice were immunized intraperitoneally (i.p.) with irradiated OVA-expressing cells to generate an anti-OVA response. On the same day, mice were depleted of pDCs via i.p. injection of PDCA depleting antibody (α-PDCA) or isotype antibody (iso). Mice were given a high-dose (HD) of gp96 24 h later. *In vivo* cytotoxicity was measured 6 days later by flow cytometry. (**b**) Percent cytotoxicity was calculated as described in Methods and is shown for each group. Data are pooled from two independent experiments and represented as mean±s.e.m. (**c**) Schema to determine Nrp1 requirement for HD gp96-mediated suppression. Mice were immunized i.p. with irradiated tumour cells. On day 7, mice were given HD gp96 (i.d.) and one i.p. dose of Nrp1 neutralizing antibody (α-Nrp1). On day 14, mice were killed for analysis. (**d**,**e**) Draining inguinal LNs were harvested for flow cytometry analysis of CD4^+^Foxp3^+^ Treg and total CD4 cells. Data are represented as mean±s.d. *ns*, not significant, *P<0.05, **P<0.01, ***P<0.001 (one-way ANOVA). HD, high dose.

We have implicated CD91^+^CD11c^+^ cells in high-dose gp96-mediated suppression of tumour immunity and shown that gp96-conditioned pDCs have the capacity to engage and form stable interactions with Treg via Nrp1. We next established a gp96-mediated suppression assay to test the role of pDCs *in vivo* (Fig. 7a). Mice were immunized with irradiated, antigen (ovalbumin, OVA) expressing or non-expressing B16 tumour cells (Group 1 and 2, respectively). OVA-specific CTL activity was measured *in vivo* by intravenous injection of SIINFEKL-pulsed or unpulsed, differentially labelled target cells into mice. In this *in vivo* assay, CTL efficiently lysed SIINFEKL-pulsed target cells in mice immunized with B16-OVA (Group 2) but not with B16 (Group 1) (Fig. 7b, Supplementary Fig. 6a). When mice were immunized with B16-OVA and treated with high-dose gp96 one day later (Group 3), CTL activity was significantly abrogated (Fig. 7b). This data is consistent with earlier observations of Treg generation by gp96 treatment^19^,^21^. pDCs were depleted with specific antibody or control IgG in this assay as described in Methods (Supplementary Fig. 6b). When pDCs were depleted (Group 4), high-dose gp96 treatment failed to abrogate CTL-mediated lysis of target cells (Fig. 7b). Control mice immunized with B16-OVA and depleted of pDCs in the absence of gp96 treatment (Group 5) still primed CTL activity comparable to Group 2. Using gp96 high-dose assay previously established, we measured the increase in Treg populations as a function of high-dose gp96 administration (Fig. 7c). Mice were immunized with B16-OVA followed by treatment with high-dose gp96. Nrp1 blocking antibody or isotype control antibody was co-administered with high-dose gp96. The increase in Treg percentages in draining lymph nodes achieved with high-dose gp96 was absent when Nrp1 antibody was co-administered (Fig. 7d). The total percentage of CD4 T cells, however, did not change (Fig. 7e). These data identify pDCs as a key APC controlling high-dose gp96-mediated suppression of protective immunity and that Nrp1 is instrumental in these observations

## Discussion

**Figure 8:**
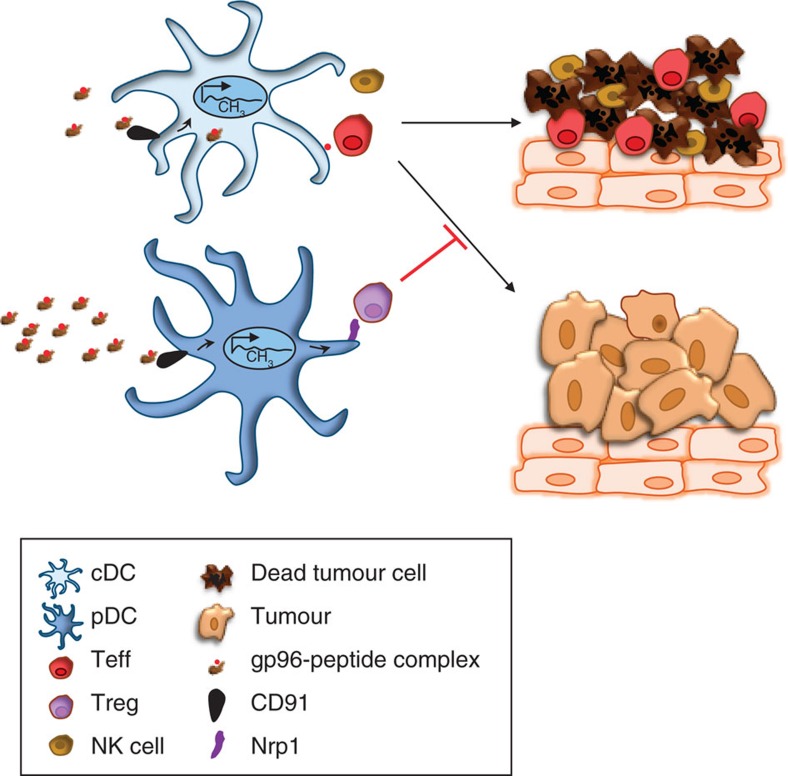
Schematic for high-dose gp96-mediated immunosuppression. At low doses, gp96 engages cDCs. Following epigenetic changes and cross-presentation of chaperoned peptides, cDCs primes Th1 anti-tumour immunity, characterized by enhanced CTL and NK cell function. At high doses gp96 engages a significantly greater percentage of pDCs. Following epigenetic remodelling, distinct from that in cDCs, pDCs stabilize interactions with Tregs via Nrp1 that suppresses ongoing Th1 responses in tumours.

Our studies have revealed, for the first time, a DC phenotype associated with a suppressive response to gp96, which can be recapitulated *in vitro*. Although CD91 is required for gp96-mediated immune responses, the engagement of CD91 by gp96 can occur on multiple APC types, ultimately determining the type of immune response that prevails. We proposed that this occurs via DNA methylome remodelling in DC, leading to altered expression of various adhesion molecules (Fig. 8). The importance of DNA methylation in driving expression of genes involved in immunity has recently begun to emerge^23^,^24^,^25^,^26^,^27^,^28^,^29^. In our study, we assessed methylome changes in CD91^+^ cells isolated from immunized mice. Although the vast majority of CD91^+^ cells are DCs and macrophages, this does not represent a pure population of target cells and must be taken into account in interpreting the results of our methyl-seq data set. However, our present study was focused on Nrp1, and we have identified a specific DC subset involved and have validated these data using bisulfite sequencing and flow cytometry in these DCs. Additional target genes identified in our screen and expressed in these particular subsets or others will need to be further investigated for their possible contributions in modulating the magnitude and nature of gp96-mediated immune responses, keeping in mind that DC-Treg interactions are mediated by a myriad of adhesion molecules^44^,^45^. Once we functionally identify the full complement of adhesion molecules, we can address the issue of Nrp1 sufficiency in the pDC-Treg interaction.

We have focused on DNA methylation here because of its emerging role in immune regulation. It has recently been shown that NF-κB can directly associate with DNMT1 in tumour cells, effectively guiding DNMT1 to its target genes^34^,^35^. The role of NF-κB signalling in response to immunogenic HSPs has been well documented by our lab and others, and is a known potentiator of gp96 responses in both cDCs^8^,^9^ and pDCs^32^. It is possible that NF-κB signalling downstream of CD91 is the catalyst for the methylome remodelling that we have observed. Although DNMT1 is primarily responsible for maintenance methylation^46^,^47^, it can also cooperate with DNMT3 to initiate *de novo* methylation^48^,^49^. We did not observe any difference in DNMT3a punctae at any time point analysed in our experiments, suggesting that DNMT1 is specifically recruited to target genes in DCs stimulated with gp96. The accessibility of DNMT target sites to the enzyme provides physical constraints on whether genes and/or regulatory elements are methylated and, therefore, can determine changes in gene expression. We observed that a regulatory element within *Nrp1* is in a closed chromatin formation (thus inaccessible) in naive cDCs but open in naive pDCs. This offers an explanation for the observed methylation patterns when stimulated with gp96, despite the similar activation patterns of DNMT1 in both cell types. Other epigenetic machinery which may be activated in response to gp96 including histone modifying microRNAs and enzymes are currently being examined in our model systems.

The majority of the DMRs that we detected in this study are associated with genes involved in cell–cell contact and adhesion. Interestingly, DNA methylation is known to have a critical role in mediating cell–cell contact within the nervous system^50^, but little is known about how DNA methylation regulates contact between immune cell populations. Intragenic methylation of *Nrp1* has been observed in human monocytes, but the consequences of this alteration on protein expression and function were not evaluated^51^. Contrary to promoter methylation, which is typically associated with decreased gene expression, methylation within the gene coding sequence (intragenic) is often a mark of active expression^39^ consistent with our observations for Nrp1 in gp96-stimulated pDCs. We performed transcription factor binding analysis on the Nrp1 DMR using Genomatix software and detected a possible binding site for neuron restrictive silencer factor (NRSF/REST). This factor is a known repressor of *Nrp1* transcription in neurons^52^ and is highly expressed in non-neuronal cells including immune cells. It is possible that methylation at this site blocks the binding of the repressor and allows for increased *Nrp1* transcription, although this remains to be formally tested.

pDCs can prime regulatory immune responses using a variety of mechanisms, including increased expression of indoleamine 2,3-dioxygenase (IDO) or upregulation of molecules associated with Treg stabilization and activation such as CTLA4 (ref. 53) and Nrp1 (ref. 37). Nrp1 expression by immature DCs and pDCs facilitates long-term interaction with Treg via homotypic Nrp1–Nrp1 interaction^36^,^37^. The role of the Nrp1-Sema4a signalling axis in maintaining Treg populations via expression of Bcl-2 and other survival factors within the tumour microenvironment has been documented^38^. Whether intercellular Nrp1–Nrp1 interactions trigger similar signalling in Treg is not known but is under active investigation. In agreement with previous reports^32^, we did not observe any increase in maturation in gp96-stimulated pDCs (Supplementary Fig. 2). These DC do, however, maintain high levels of major histocompatibility complex II expression, which is critical for effective Nrp1–Nrp1 interaction with CD4^+^ Treg. Although the circumstances in which antigen presentation occurs on pDCs is contested, it has been reported that the triggering of various endocytic receptors on pDCs results in the increased capacity of these cells to present antigen^54^. Endocytosis of gp96-CD91 complexes on pDCs could allow for presentation of self antigen(s) chaperoned by gp96. We observed that high-dose gp96-mediated suppression is dependent on pDCs, since their depletion results in maintenance of CTL lysis of target cells (Fig. 7b). It was previously shown by our lab that gp96 is passively drained to the subcapsular sinus of lymph nodes, where it is taken up by DCs^31^. It is possible that higher doses of gp96 are required to diffuse to pDCs, which normally reside within the T-cell zone or pDCs are less sensitive to gp96 and require a higher dose for activation. In addition to Treg activation, gp96 stimulated pDCs may also have a role in limiting cDC activation. Previous work from our lab has shown that, at least *in vitro*, gp96-stimulated pDCs abrogates the ability of cDCs to mediate pro-inflammatory responses^32^. Considering that both DC types interact with gp96 after high-dose immunization, pDCs offer a two-pronged approach to inhibition of Th1 responses.

In this study, we have examined an immunosuppressive role for exogenous gp96, introduced by vaccination. We postulate that these mechanisms may also be applicable when gp96 is inadvertently released from aberrant cells, including tumour cells and infected cells^55^. Intracellular gp96 has also been shown to play an important role in regulating Treg function via its role in chaperoning a number of immune-related proteins including glycoprotein A repetitions predominant^56^, a membrane docking protein for latent-membrane associated TGF-β, integrins and TLRs^56^,^57^,^58^. Thus, depending on its localization, gp96 has a number of divergent mechanisms to control Treg function; extracellularly and indirectly via CD91 on pDCs as shown here, or intracellularly, relying on its ability to chaperone immune-related proteins^56^,^57^,^58^. On a cellular level, these two mechanisms may reflect a population-based or individualized approach for Treg control, respectively.

Following demonstrable efficacy in mouse models of cancer^11^, low-dose gp96 is currently in clinical trials for the immunotherapy of cancer^14^,^15^,^16^. High-dose gp96 therapy of autoimmune disease has only been documented in mice, but clinical trials are anticipated in the future. Our studies provide a better understanding of how dichotomous immune responses may be initiated and regulated based on the nature and dose of an administered immunogen.

## Methods

### Mice

C57BL/6 mice were obtained from Jackson Laboratories (Bar Harbor, ME) and housed in DLAR at the University of Pittsburgh post-purchase. CD91^f/f^CD11c^cre^ mice (on the C57BL/6 background) were generated in our DLAR facility and have been previously characterized^30^. For tumour growth experiments, both male and female mice were used. All mice were 6–8-weeks old. All experimental procedures were approved by the University of Pittsburgh, Institutional Animal Care and Use Committee and performed in compliance with its guidelines.

### Cells

To obtain BMDCs, bone marrow cells were isolated from the femurs of female C57BL/6 and cultured in complete RPMI (10% heat-inactivated fetal bovine serum)(GIBCO). Cells were cultured in RPMI with granulocyte–macrophage-colony-stimulating factor (Fisher Scientific). Media was supplemented after 3 days of culture and loosely adherent cells were harvested 6–7 days following initial plating. pDCs were isolated from mouse spleens using anti-PDCA1 microbeads according to the manufacturer's protocol (Miltenyi Biotec) and cultured in complete RPMI. Approximately 5–10% of CD11c^+^CD11b^−^ are B220^+^PDCA^+^ in the inguinal lymph nodes and spleen. cDCs were isolated from spleen using magnetic beads for CD11c^+^ cells. D122 was obtained from Dr M. Feldman (Department of Immunology, Weizmann Institute of Science, Rehovot, Israel). B16.F0 were obtained from ATCC. B16.F0-OVA were obtained from Dr L. Falo (Department of Dermatology, University of Pittsburgh, Pittsburgh PA). Cell lines were negative for mycoplasma by PCR as tested by the Research Animal Diagnostic and Investigative Laboratory (RADIL) (University of Missouri). D122 and B16.F0 tumour cells were cultured in complete DMEM (10% FBS). B16.F0-OVA cells were cultured in DMEM supplemented with 2.5 mg ml^−1^ G418. PECs were freshly harvested from the peritoneum of mice using PBS and cultured overnight for the adherent population. mAb927 hybridoma was a gift from Dr Shlomchik (Department of Immunology, University of Pittsburgh, Pittsburgh PA).

### Cell culture

For all pDC stimulation assays *ex vivo*, 5 × 10^5^ cells were plated in round bottom plates with 10 μg ml^−1^ or 200 μg ml^−1^ gp96, or equivalent volume of PBS. cDCs were plated at 5 × 10^5^ cells per well with 200 μg ml^−1^ gp96 in round bottom plates. For 5-azaC treat pDC generation, mice were administered 5 mg kg^−1^ 5-azaC in a total volume of 200 μl intraperitoneally (i.p.), and pDCs were isolated by magnetic cell sorting after 72 h. For DNMT western blotting, nuclear extracts were isolated using Epiquik Nuclear Extraction Kit (Epigentek). DNMT1 (clone 60B122.1; Epigentek) and Lamin B1(clone L-5; Invitrogen) antibodies were used in immunoblots and were developed using X-ray film. Bands were quantified using ProteinSimple technology. For bisulfite sequencing, cells were harvested in Puregene cell lysis buffer (Qiagen) before DNA extraction. The mAb927 hybridoma was cultured in complete RPMI (10% FBS). Cells were serum starved for 3 days, supernatants were harvested, dialysed, and run through a Melon Gel column (Thermo Fisher) for purification of the PDCA antibody.

### Purification of HSPs

In all experiments, gp96 was purified from mouse livers. Mouse livers were harvested and homogenized with a blender to a single cell suspension. Protein was stepwise precipitated out of an ultracentrifugation (100,000*g*) supernatant to 80% saturation stepwise. Protein was passed over immobilized Concanavalin A columns. Bound fraction was eluted with 10% mannose and buffer-exchanged with PD10 columns into 0.005 M sodium phosphate solution containing 0.3 M NaCl. This was applied to equilibrated DEAE columns. Bound fraction was eluted with 0.7 M NaCl. Fraction 3–5 contained gp96 as assessed by SDS–polyacrylamide gel electrophoresis and immunoblotting. We fully characterized all gp96 preparations; purity of gp96 preparations was assessed by SDS–polyacrylamide gel electrophoresis gp96 and was determined to be a homogenous 96kDa protein (Supplementary Fig. 7a). The protein was recognized by a monoclonal gp96 antibody (clone 9G10, Enzo Life Sciences) (Supplementary Fig. 7b). Further, gp96 retained its enzymatic ATPase activity (Supplementary Fig. 7c). ATP hydrolysis was measured using the ATPase/GTPase Activity Assay kit (Sigma). Gp96 (10 mg per well) was incubated for indicated period of time with ATP at 37 °C in a 96-well plate. Malachite green was added and absorbance was read at 600 nm. ATP hydrolysis was calculated using a standard curve.Gp96 was determined to be free from endotoxin contamination by LAL assay and western blot (Supplementary Fig. 7). Gp96 concentrations were determined by the Bradford assay. All immunizations were delivered intradermally (i.d.) in a final volume of 100 μl PBS (1 μg- low dose; 10 μg- high dose). For all *in vitro* assays using cDCs or pDCs, cells were cultured with gp96 at the indicated concentration (10 μg ml^−1^- low dose; 200 μg ml^−1^- high dose) or an equivalent volume of PBS. Gp96 was labelled with A488 as previously described^31^.

### *In vivo* tumour growth assay

For active immunization, tumour cells were irradiated (6,000 rad) and 1 × 10^6^ cells were injected subcutaneously as indicated. One week later, mice were injected with PBS, low-dose gp96, high-dose gp96, or MSA. One week following treatment, mice were subsequently challenged i.d. with 5 × 10^4^ tumour cells and monitored for tumour growth by measurement on two axes. Ethical approval by Institutional Animal Care and Use Committee allowed tumour growth to 2cm in diameter and this limit was strictly adhered to; mice were killed when tumours were 1.5 cm in any one diameter. Group size was determined by power analyses in each experiment.

### MBD methyl-sequencing and analysis

Mice were immunized i.d. with low-dose or high-dose gp96 or with PBS and killed after 18 h. Draining lymph nodes were harvested and CD91^+^ cells were isolated by FACS using a FACSAria instrument (BD Biosciences). DNA was purified, sonicated to an average size of 300 bp using a Bioruptor (Diagenode), and enriched for methylated sequences using MBD purification using the MethylMiner Enrichment Kit (Invitrogen). Precipitated DNA was analysed for quality assurance before sequencing. Methylated fragments were sequenced using an Ion TorrentN sequencer (Life Technologies) at the Genomics and Proteomics Core (University of Pittsburgh, PA). The samples were sequenced to an average of 45 million reads. Raw sequencing reads (fastq format) were mapped to the mouse genome (mm9) using Bowtie 2, which can account for large gaps in sequencing reads. Only nonredundant and uniquely mapped reads were used for subsequent analysis. Methyl peaks were assigned using Model-based Analysis for ChIP-Seq (MACS) software^59^ with PBS treated mice as a reference sample. Peaks were merged based on overlapping and book-end peaks, and coverage was calculated using BEDtools merge version 2.17.0. Peaks were annotated with the annotation provided by UCSC using a custom Perl wrapper and Tabix^60^. Peaks within 2,000 bp of a coding sequence were reported with annotation. Comparison of peaks and identification of DMRs was performed using the Genome Expression Data Analyser software and Efficiency Analysis tool developed by the University of Pittsburgh Bioinformatics Core^61^,^62^,^63^. DMRs were visualized using the UCSC Genome Browser and Genomatix software. Global distribution of intragenic DMRs (exon, intron, promoter) was performed using Genomatix Genome Analyser (Genomatix Software GmbH). Distribution across TSS and CpG islands was determined using SeqMonk (Babraham Bioinformatics). Transcription factor binding analysis was performed using Genomatix software. Genetrail^64^ was used to test for gene ontology analysis using default parameters and Ensembl genes as background for enrichment. Results were corrected for multiple testing using Bonferroni adjustment. Expression of target genes by APCs was verified using the Immunological Genome project consortium (ImmGen).

### Quantitative real-time PCR

Cells were lysed in cell lysis buffer and RNA was extracted using RNeasy Mini Kit (Qiagen) according to manufacturer protocol. RNA was reverse transcribed using the High Capacity cDNA Reverse Transcription kit (Applied Biosystems). Cycling conditions for Nrp1 qPCR were 10 min at 95 °C, followed by 40 repeats of 95 °C for 15 s and 58 °C for 45 s. Primers used were Nrp1 forward: 5′-CCGGTTACCCTCATTCTTAC-3′, Nrp1 reverse: 5′-CAGTCTCTGTCCTCCAAATC-3′. In the real time PCR, cDNA was amplified using Power SYBR Green PCR master mix (Applied Biosystems).

### Bisulfite PCR

For pDC experiments *in vitro*, splenic pDCs were isolated by magnetic sorting and pulsed with 200 μg ml^−1^ gp96. Cells were harvested after 6 h. Genomic DNA was bisulfite converted with the EZ Lightning DNA Conversion Kit according to the manufacturer's protocol (Zymo Research). Methylated and unmethylated DNA controls were spiked into each sample to control for completeness of the bisulfite conversion. Bisulfite-converted DNA was amplified by PCR (F primer: 5′-TTGTATTGAGGTATATAAAGTTGGTA-3′, R primer: 5′-AATTCAAAAACACAAATTTCTCTCC-3′; generated using MethPrimer^65^) and the amplicons were cloned using TOPO TA vectors (Invitrogen). Colonies were isolated and minipreps were performed to isolate DNA. The DNA was then sequenced for Nrp1. Both CpG and non-CpG were considered in analyses.

### Chromatin accessibility assay

Naive pDCs and total CD11c^+^ DCs were isolated from C57BL/6 spleens using anti-PDCA and anti-CD11c isolation kits (Miltenyi Biotec) according to the manufacturer protocols. Chromatin was immediately purified and digested from isolated DCs using the EpiQuik chromatin accessibility assay kit (Epigentek). Chromatin preps from each sample were split in two, and were treated with nuclease (Nse) mix or left untreated. DNA was amplified using quantitative PCR using primers specific for the Nrp1 region identified from the methyl-seq/bisulfite seq screen. Fold enrichment was calculated using the formula: FE=2̂(NseCT-noNseCT) × 100%. Samples were validated using positive and negative control primers provided by Epigentek.

### Microscopy and analysis

Non-confluent cells (BMDCs, cDCs, pDCs and PECs) were grown overnight on coverslips at 37 ^o^C then pulsed with PBS or gp96 for 6 h. Cells were then washed, fixed in 2% paraformaldehyde for 15 min, permeabilized with triton X-100 for 15 min, blocked with 2% bovine serum albumin (BSA), and stained in 0.5% BSA with primary antibody anti-DNMT1 (clone 60B122.1, Epigentek) at a dilution of 1:100. Secondary antibodies were conjugated to Cy3, Cy5 or AF488. F-actin was stained with phalloidin-488 and nuclei were stained using Hoescht stain. All images were captured using an Olympus FV1000 confocal microscope. DNMT puncta were quantified using analyses developed by the Center for Biologic Imaging (University of Pittsburgh, PA), which calculates punctae intensity based on intensity threshold using NIS Elements (Nikon). The number of puncta per field of view were normalized to the total number of cells in that field of view. For cardamonin studies, PECs were treated with high-dose gp96 in the presence of 10 mM cardamonin or equivalent amount of DMSO. Cells were stained for DNMT after 6 h incubation at 37 °C.

### Live cell imaging and analysis

Splenic pDCs were plated at 5 × 10^5^ cells per dish in 35-mm collagen-coated glass-bottom culture dishes (MatTek) in 10% FBS complete RPMI. Cells were pulsed with PBS or low-dose or high-dose gp96 for 18 h. CD4^+^CD25^+^ Tregs or CD4^+^CD25^−^ Tconv were isolated from spleen using Treg isolation kit (Miltenyi) and labelled with CellTracker Red dye before addition to the culture and imaging. Nrp1 blocking monoclonal antibody (clone 761704; RnD Systems) or rat IgG2a isotype antibody (clone eBR2a; eBioscience) were added 2 h before imaging at a final concentration of 10 μg ml^−1^. Antibody clones and concentrations were based on previously established protocols^38^. Cells were imaged on a Nikon A1 inverted microscope (Nikon) using a 40X objective. Images were captured every 5 min for a total of 1 or 2 h. Videos were analysed using NIS Elements (Nikon) and ImageJ^66^ with Manual Tracking plugin. For interaction time analysis, total contact duration per Treg/Tconv detected from videos was calculated based on the number of frames in which a Treg/Tconv came in contact with a pDC.

### Flow cytometry

Conjugated antibodies included: APC/Cy7 anti-CD11c (clone HL3, BD), PerCP-Cy5.5 anti-CD11b (clone M1/70, BD), APC anti-PDCA-1 (clone eBio927, eBioscience), BV421 anti-Nrp1 (clone 3E12, BioLegend), FITC anti-B220 (clone RA3-6B2, BD), anti-Lrp1 (clone 5A6, Abcam), PerCP-Cy5.5 anti-CD4 (clone RM4-5, BD) and APC anti-Foxp3 (clone FJK-16s, eBioscience). Surface marker staining was performed in staining buffer (0.5% BSA+0.1% sodium azide in 1X PBS) at a 1:100 dilution for 20 min on ice followed by washing. Foxp3 staining was performed using overnight fixation and specific staining kit (eBioscience) after staining for surface markers. CD91 staining was performed at 4 ^o^C for 30 min following a 15 min fixation with BD fix/perm buffer. All experiments were performed using BD LSR II and BD LSRFortessa instruments and analysed using FlowJo software. Gating strategies for cDCs, pDCs and T cells are provided in Supplementary Fig. 8.

### *In vivo* T-cell suppression assay

On day 1, C57BL/6 mice were immunized with 10^6^ irradiated (6,000 rad) B16 or B16-OVA cells i.p. in 200 μl PBS. Mice were allowed to rest 4–6 h then administered anti-PDCA (clone mab927) or control rat isotype IgG2b (clone A95-1; BD) in 200 μl PBS for depletion of pDCs. Each mouse received 400 μg i.p., consistent with previous studies using this antibody for *in vivo* depletion^67^. pDC depletion was measured by the percent of pDCs present in the blood at the time of gp96 immunization (Supplementary Fig. 6b), which confirmed at least ∼90% depletion at this time point. One day later, mice were given high-dose gp96 i.d. On day 7, C57/BL6 mouse splenocytes were divided, pulsed with SIINFEKL peptide, and CFSE labelled. Unpulsed (CFSE-low) and SIINFEKL-pulsed (CFSE-high) were mixed at a 1:1 ratio and 1–2 × 10^7^ cells were injected intravenously via tail vein. Recipient mice were killed 5 h later and analysed by flow cytometry for lysis of the fluorescently labelled target population. Percent cytotoxicity was calculated: (1-(input ratio/sample ratio)) × 100.

### *In vivo* Nrp1 neutralization assay

On day 1, C57BL/6 mice were immunized with 10^6^ irradiated (6,000 rad) B16-OVA cells i.p. in 200 μl PBS. Seven days later, mice were given high-dose gp96 i.d. in 100 μl PBS (or PBS only as control). On the same day, mice also received 100 μg Nrp1 blocking antibody (clone 761704, RnD Systems) or 100 μg rat IgG2a isotype antibody (clone eBR2a, eBioscience) via the i.p. route. Route and dose of Nrp1 antibody is based on previous reports using this antibody^38^. One week later, all mice were killed and draining lymph nodes were harvested for flow cytometry. The percent of Foxp3+ T cells was quantified.

### Statistical analyses

Statistical analyses were performed using 2-tailed *t*-test for comparison between two variables or one-way analysis of variance (ANOVA) with Tukey multiple corrections tests for comparison between three or more variables. For tumour growth studies, ANOVA of area under curve analysis was used. Statistical significance was defined as *P*<0.05. Graphs are presented as mean±standard error of mean (s.e.m.) or standard deviation (s.d.) as noted. Statistical analyses were performed using Prism software (GraphPad, La Jolla, CA). ns, not significant; *P<0.05, **P<0.01, ***P<0.001.

### Data availability

All relevant data are available from the authors.

## Additional information

**How to cite this article:** Kinner-Bibeau, L. B. *et al*. HSPs drive dichotomous T-cell immune responses via DNA methylome remodelling in antigen presenting cells. *Nat. Commun.* **8,** 15648 doi: 10.1038/ncomms15648 (2017).

**Publisher's note:** Springer Nature remains neutral with regard to jurisdictional claims in published maps and institutional affiliations.

## Supplementary Material

Supplementary Information Supplementary Figures and Supplementary Tables
