# Supplementary material for: HSPs drive dichotomous T-cell immune responses via DNA methylome remodelling in antigen presenting cells
Source: Nat Commun. 2017 May 31;8:15648. doi: 10.1038/ncomms15648 (PMC5460028; doi:10.1038/ncomms15648)
Supplement: Supplementary Information — Supplementary Figures and Supplementary Tables [file ncomms15648-s1.pdf]

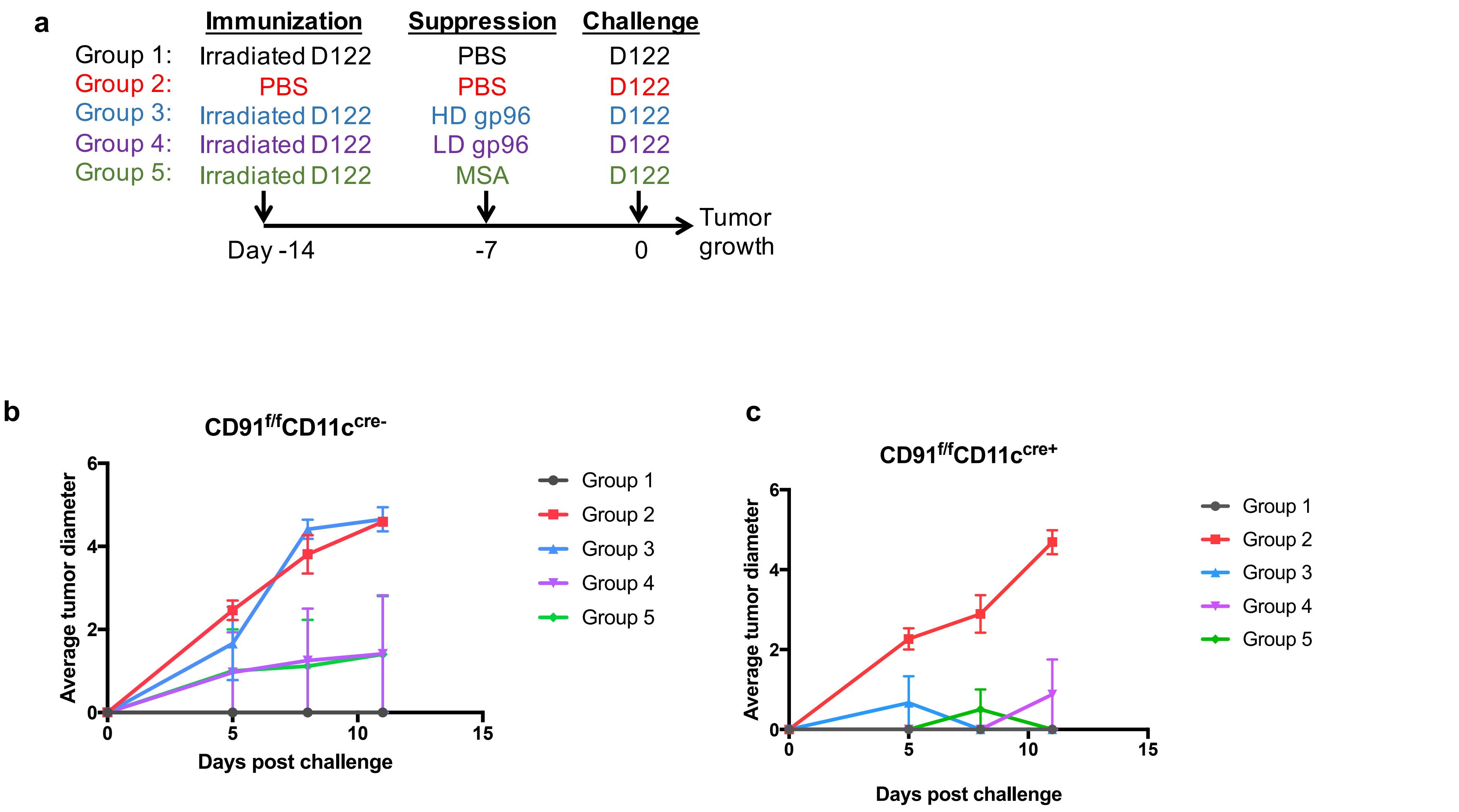

Supplementary Figure 1. Gp96 elicits immune suppression at high dose but not low dose. (**a**) Schema of experimental groups to test dose effects of gp96 on immune suppression. Mice were immunized with irradiated tumor cells and treated with low or high dose gp96 or with a control protein, mouse serum albumin, after 7 days. Immunity was measured by rejection of a tumor challenge on day 0. Tumor diameter was measured by two perpendicular axes using calipers. Tumor growth is shown for CD91$^{f/f}$ (**b**) and CD91$^{f/f}$CD11c^{cre} (**c**) mice. Data are represented as mean±s.e.m., n=5/group. HD=high dose, LD=low dose, MSA=mouse serum albumin

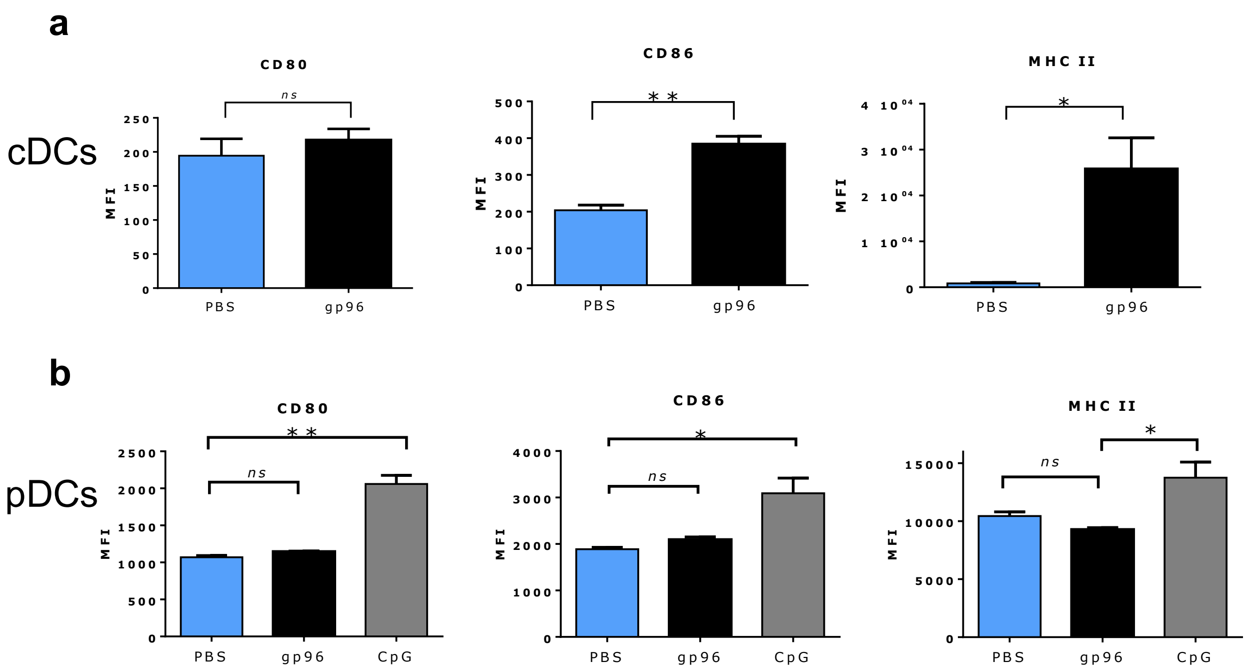

Supplementary Figure 2. cDCs undergo maturation in response to gp96, whereas pDCs retain an immature phenotype. cDCs (**a**) or pDCs (**b**) were stimulated with HD gp96 for 24 hours *in vitro* and then analyzed for maturation markers CD80, CD86, and MHC II by flow cytometry. CpG DNA-stimulated pDCs were included as a control for pDC maturation. Average MFI is shown for each marker. Data are represented as mean±s.d., n=3/group. *ns* not significant, *P<0.05, **P<0.01 (Student's t test used in panel a; one-way ANOVA used in panel b).

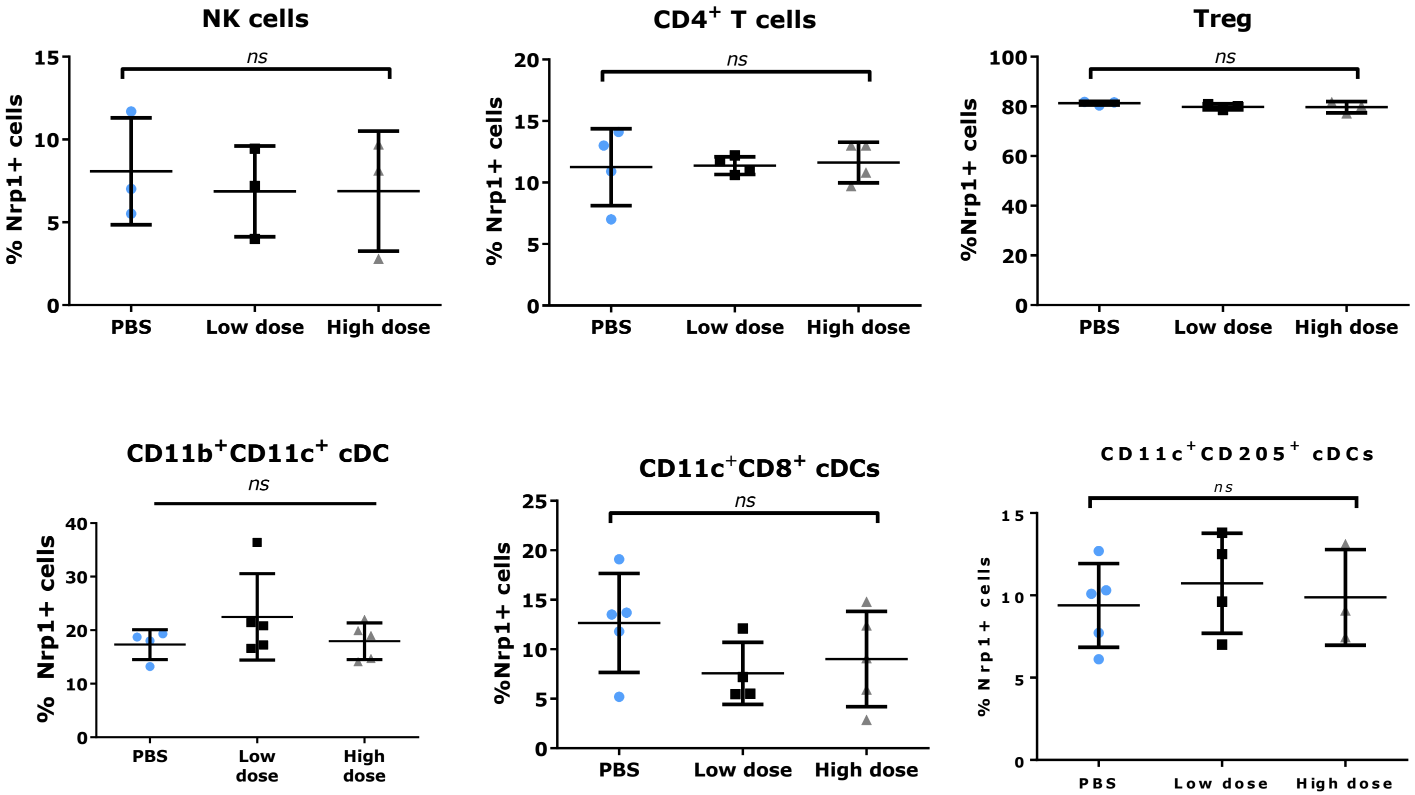

Supplementary Figure 3. Cell surface Nrp1 expression does not change on majority of hematopoietic cells following gp96 immunization. WT mice were immunized with LD or HD gp96 or given PBS. Lymph nodes were harvested after 18 hours and stained for NK cells (NK1.1), $CD4^+$ T cells (CD3, CD4), Treg (CD3, CD4, Foxp3), and DC subsets (CD11b, CD11c, CD8, CD205). Cells were analyzed for changes in percentage of Nrp1 expressed in respective gates. Data are represented as mean±s.d. *ns* not significant (one-way ANOVA).

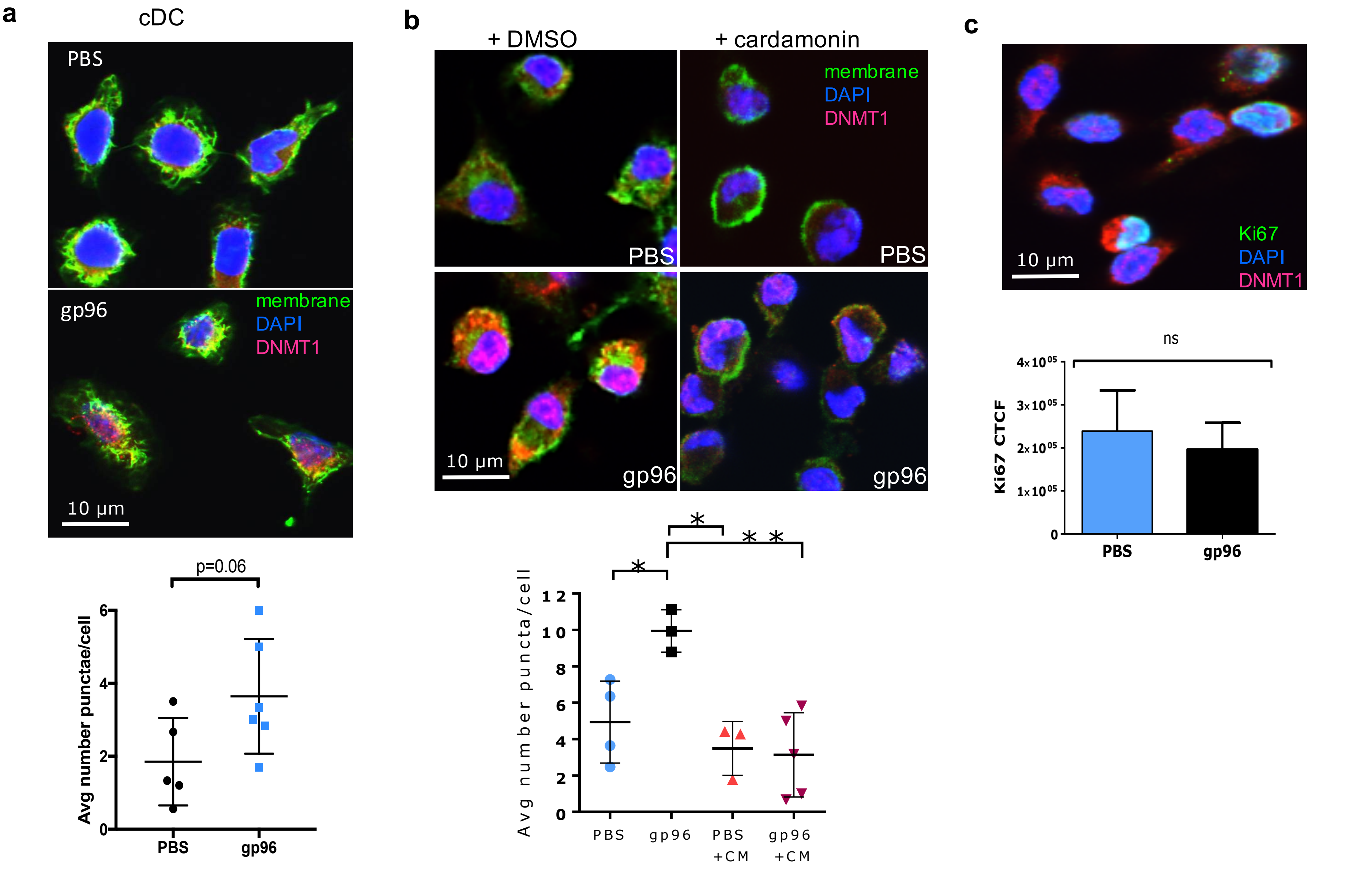

Supplementary Figure 4. DNMT1 punctae formation in gp96-stimulated DCs is NF-kB dependent. (**a**) Splenic cDCs were harvested from naïve mice, treated with HD gp96, and analyzed by confocal microscopy after 6 hr. DNMT punctae were quantified as described in Methods. (**b**) Cells were treated with HD gp96, in the presence of 10 µM NF-kB inhibitor cardamonin (DMSO as negative control). DNMT1 punctae analysis was performed by confocal microscopy after 6 hr. (**c**) BMDCs were treated as in A and stained with DNMT1 and Ki67 antibodies to analyze Ki67 expression and location. Bar graphs on the right are the average of 2 experiments. Data are represented as mean±s.d. *ns* not significant, *P<0.05, **P<0.01 (Student's t test used in panels a and c; one-way ANOVA used in panel b).

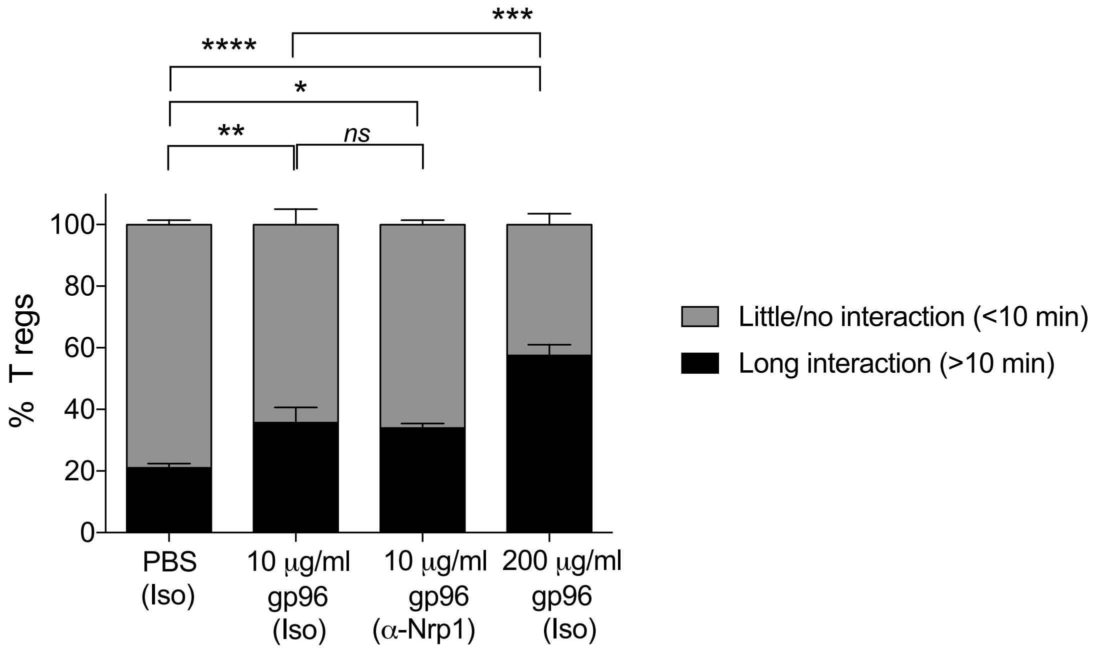

Supplementary Figure 5. Duration of pDC-Treg interaction increases with increasing treatment dose of gp96. pDCs were treated with low or high dose gp96 for 18 hr. As described in Methods, pDCs were also treated with a-Nrp1 blocking antibody or isotype control and co-cultured with labeled Treg. The percentage of cells which had undergone long term interactions in each group was calculated and is shown. Data are represented as mean±s.d. *ns* not significant, *P<0.05, **P<0.01, ***P<0.001 versus control (ANOVA).

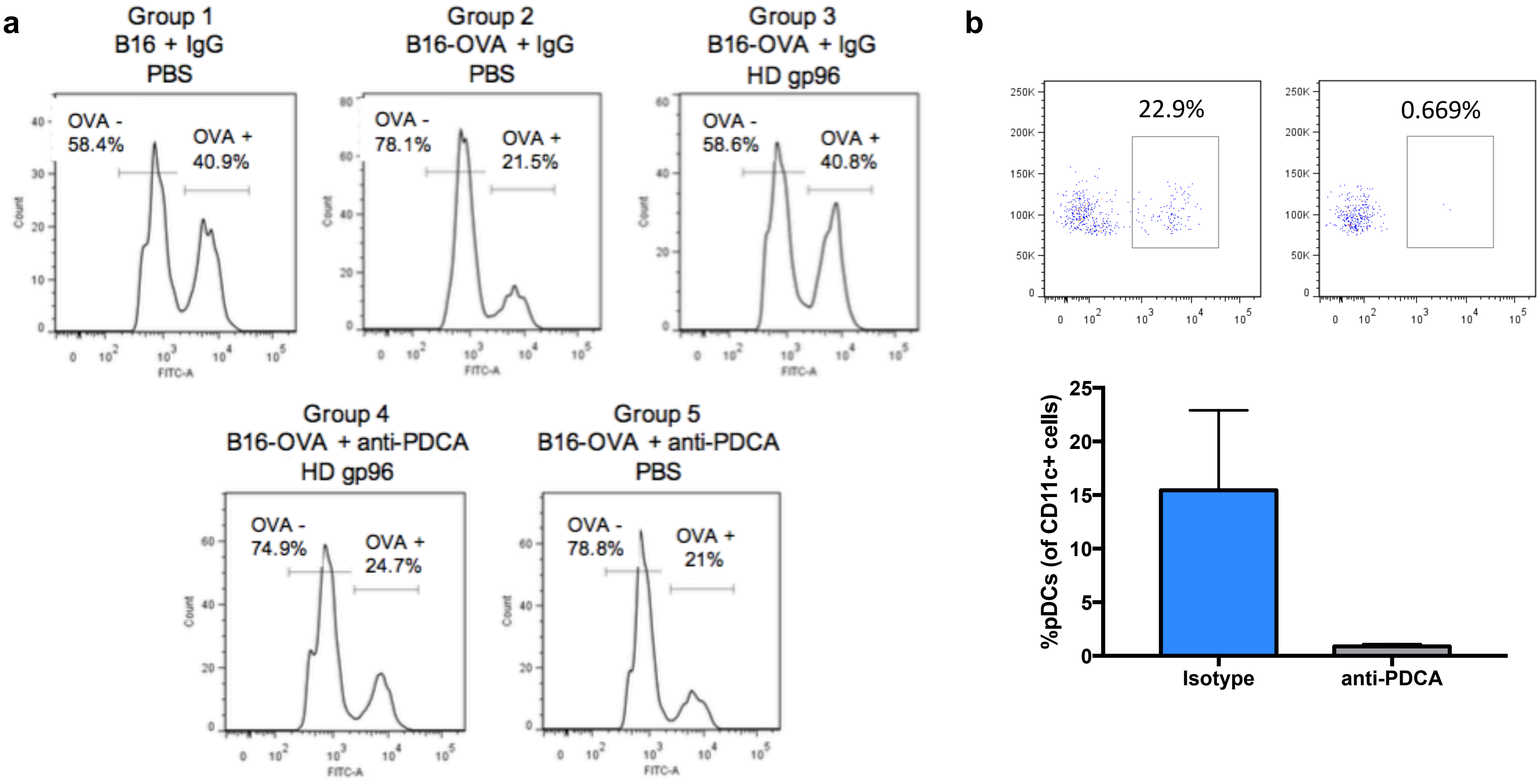

Supplementary Figure 6. HD gp96 promotes suppression of anti-OVA CTL *in vivo* in a pDC-dependent manner. (**a**) Shown are representative flow plots for CFSE-labeled SIINFEKL-pulsed target splenocytes (CFSE-high) and unpulsed non-target splenocytes (CFSE-low) for each treatment group in Figure 7A. (**b**) Mice were treated with a-PDCA depleting antibody or isotype control antibody i.p. (400 µg/mouse). pDC depletion was monitored in blood 24 hr later by flow cytometry. Data are represented as mean±s.d.

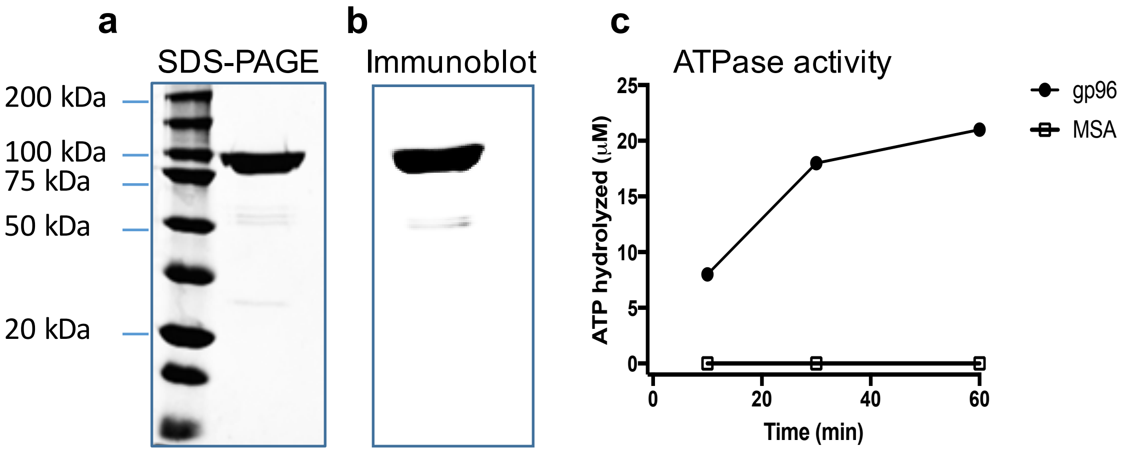

Supplementary Figure 7. Characterization of purified murine gp96. Gp96 preparations were tested for purity and biochemical activity to ensure quality of protein. (**a-b**) gp96 was analyzed by 10% SDS-PAGE following staining with Coomasie blue, and by immunoblotting with a gp96 specific monoclonal antibody. (**c**) ATP hydrolysis by gp96 was assessed. Gp96 was incubated at 37°C for indicated time period with ATP. Following addition of malachite green, μM ATP hydrolyzed was calculated from a standard curve. MSA was used as a negative control.

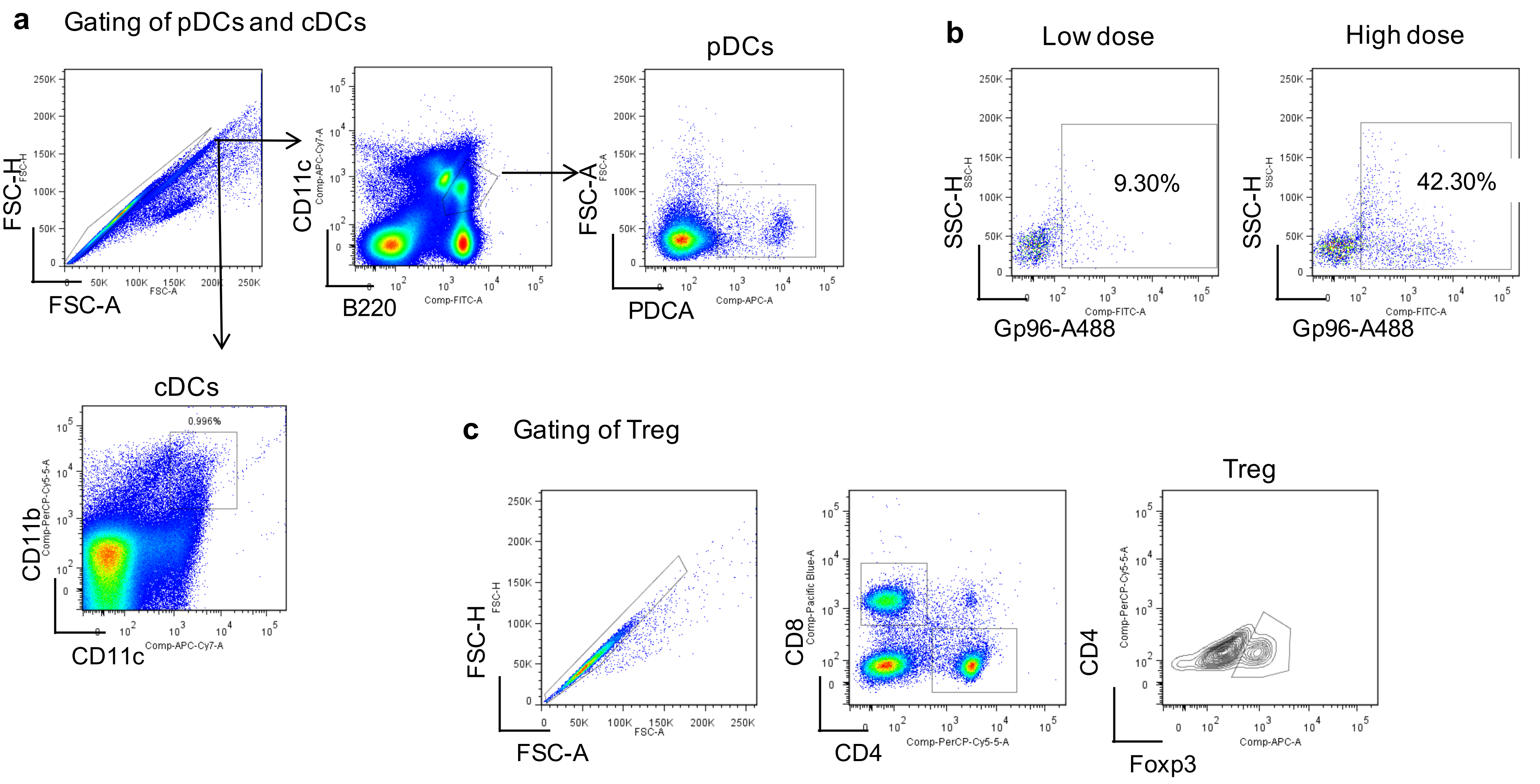

Supplementary Figure 8. Flow cytometry gating strategy for pDCs, cDCs, and T cells. Gating strategy for cell types identified throughout manuscript. (**a**) pDCs were identified by expression of CD11c (intermediate), B220, and PDCA. cDCs were identified by expression of CD11c and CD11b. (**b**) Using a similar gating strategy shown in (a), $gp96_{A488}$ positive cells were identified by FITC fluorescence. (**c**) Treg were identified by expression of CD4 and Foxp3.

Sample	Total Reads	Filtered Reads	% Lost	% Mapped	# Mapped
PBS	36439249	6737640	18.49	99.51	6704382
Low dose	46698860	7339383	15.72	99.57	7307944
High dose	55685332	11229658	20.17	99.65	11190094

Supplementary Table 1. Methyl-seq sample mapping statistics

HD>LD (29 targets)

Gene name	Aliases	RefSeq accession	Chromosome	Peak start	Peak end	HD coverage†	LD coverage†	Delta (HD-LD)††	APC expression (Immgen)	Published works on DC-T cell interaction
Magi2	ACVRI1	NM_001170746	chr5	18985287	18985930	706.93	290.78	416.15	-	
Anks1b	AIDA-1B	NM_001128086	chr10	89716364	89717299	829.69	456.22	373.47	-	
Cep128		NM_181815	chr12	92462122	92462553	413.38	56.47	356.91	cDC, pDC, mo	-
Apool	Mic27	NM_026565	chrX	109482705	109483140	428.42	94.6	333.82	cDC, pDC, mac	
Adamtsl3		NM_001190374	chr7	89618915	89619291	467.33	157.3	310.03	-	
Smo		NM_176996	chr6	29697352	29699387	534.21	295.08	239.13	mo, LC, mac	-
Nlgn1	NL1	NM_001163387	chr3	25820638	25821459	437.08	216.12	220.96	cDC, mo LC, mac	-
Pisd-ps2		NR_003519	chr17	3081229	3083292	552.73	375.78	176.95	cDC, pDC, mo LC, mac	-
Maoa		NM_173740	chrX	16247784	16248261	242.09	103.15	138.94	LC, mac	-
Unc13c		NM_001081153	chr9	73675204	73675598	320.61	195.01	125.6	-	
Gstcd		NM_026231	chr3	132728372	132728926	196.14	93.23	102.91	cDC, mac	-
Bmper	CRIM3	NM_028472	chr9	23094351	23094641	101.46	0	101.46	cDC, pDC	-
Olfr593		NM_146380	chr7	110358292	110358906	255.6	156.56	99.04	-	
Gphn		NM_145965	chr12	79375574	79376162	256.73	160.44	96.29	cDC, pDC, mo LC, mac	-
Gtf2h2		NM_022011	chr13	101241094	101241462	161.11	68.55	92.56	cDC, pDC, mo LC, mac	-
Ston2	STN2,STNB	NM_175367	chr12	92981055	92982529	86.76	0	86.76	cDC, mac	-
Meis1		NM_010789	chr11	18852461	18852694	81.39	0	81.39	cDC, pDC, mo LC, mac	-
Gpr98	ADGRV1	NM_054053	chr13	81551933	81552441	77.93	0	77.93	-	
Myo3b		NM_177376	chr2	69875218	69875746	76.25	0	76.25	-	
Cpxm2		NM_018867	chr7	139307057	139307716	76.23	0	76.23	LC	-
Wdtc1	DCAF9	NM_199306	chr4	132873894	132874522	75.33	0	75.33	cDC, pDC, mo LC, mac	-
Gabbr2		NM_001081141	chr4	46807144	46807883	74.68	0	74.68	cDC, pDC, mo LC, mac	-
Cdhr3		NM_001024478	chr12	33760550	33761112	73.54	0	73.54	cDC	-
Nrp1	CD304	NM_008737	chr8	130909774	130910078	72.41	0	72.41	cDC (low), pDC (high), mo (high)	Sarris et al, 2008; Tordjmann et al; Delgoffe et al, 2014
Ccl11	Eotaxin-1	NM_011330	chr11	81877343	81877624	72.41	0	72.41	cDC, pDC, mo LC, mac	-
Shroom3	APXL3	NM_015756	chr5	93190064	93190518	72.13	0	72.13	cDC, pDC, mo LC, mac	-
Nell1		NM_001037906	chr7	57580690	57580936	61.26	0	61.26	-	
Kctd16	M-EAG	NM_026135	chr18	40538033	40538370	61.26	0	61.26	-	
Kcnh1		NM_001038607	chr1	194023294	194023710	173.55	113.32	60.23	LC	-

LD>HD (26 targets)

Gene name	Aliases	RefSeq accession	Chromosome	Peak start	Peak end	HD coverage†	LD coverage†	Delta (HD-LD)††	APC expression (Immgen)	Published works on DC-T cell interaction
Fam172a		NM_138312	chr13	77919765	77920290	367.91	742.72	-374.81	cDC, pDC, mo LC, mac	-
Tbc1d9	MDR1	NM_001111304	chr8	85752214	85755805	94.18	401.81	-307.63	cDC, mo, LC, mac	-
Hpse2	UFS	NM_001081257	chr19	43313714	43314089	764.69	1053.75	-289.06	-	
Sgcz		NM_145841	chr8	38696342	38696718	367.91	648.16	-280.25	-	
Tmem232		NM_001008973	chr17	65629264	65629806	154.26	362.48	-208.22	-	
Stam2	HBP	NM_019667	chr2	52551744	52558220	0	163.43	-163.43	cDC, pDC, mo LC, mac	
Dach2		NM_001142570	chrX	110665377	110665793	53.44	207.81	-154.37	-	
Tmem132c		NM_175432	chr5	127752052	127752513	171.25	323.82	-152.57	cDC	-
Col19a1		NM_007733	chr1	24452000	24452373	146.76	297.98	-151.22	-	
Pik3c2g		NM_207683	chr6	139870145	139870612	77.18	228.28	-151.1	-	
Itgbl1		NM_145467	chr14	124343810	124344457	197.85	347.7	-149.85	-	
Nbea	LYST2	NM_030595	chr3	55924530	55925050	246.34	393.42	-147.08	cDC, pDC, mo, mac	-
Unc80		NM_175510	chr1	66728690	66729004	56.57	196.22	-139.65	-	
Klra2	Ly49b	NM_001170851	chr6	131177385	131177785	101.61	234.86	-133.25	mo, mac	-
Sntg1	SYN4	NM_027671	chr1	8708056	8708514	166.38	297.98	-131.6	-	
Cntn5	NB2	NM_001033359	chr9	10173900	10174397	131.91	242.32	-110.41	-	
Pip5k1a		NM_008847	chr3	94896304	94896722	0	109.78	-109.78	cDC, pDC, mo LC, mac	-
Lama2		NM_008481	chr10	27302267	27302697	0	108.28	-108.28	-	
Inpp5a		NM_001127363	chr7	146703630	146703951	0	107.37	-107.37	cDC, pDC, mo LC, mac	-
Gabrg3		NM_008074	chr7	64227382	64227879	380.3	482.22	-101.92	cDC, mac	-
Kir3dl1	NKB1	NM_177749	chrX	133051381	133052112	367.63	468.62	-100.99	-	
Ube2e2		NM_144839	chr14	19535822	19536247	374.14	461.11	-86.97	cDC, LC, mac	-
Ncam1	CD56	NM_001113204	chr9	49463833	49464363	0	86.52	-86.52	mac	-
Cadps2		NM_001252105	chr6	23410121	23410681	0	73.7	-73.7	mac	-
Ctnnd2	NPRAP	NM_008729	chr15	30589483	30589996	0	67.3	-67.3	cDC, pDC, LC	
Rbfox1	FOX1, A2BP	NM_021477	chr16	7242782	7243199	0	64.83	-64.83	cDC, pDC, mo LC, mac	

Supplementary Table 2. List of methyl-seq DMRs. Gene names and RefSeq accession numbers of all intragenic DMRs are provided along with chromosome location and start/end location of detected peaks. Each gene was also compared to the Immgen database for expression in various APC types and listed accordingly. †: Coverage values reflect the relative abundance of reads at a given region. For coding genes, this reflects the average coverage of the total sum of exons. ††: Delta is the difference between high dose and low dose coverage values. cDC=conventional DC, pDC=plasmacytoid DC, LC=Langerhans cell, mo=monocytic, mac=macrophage, HD=high dose, LD=low dose.

HD>LD				
**Subcategory name**	**pval**	**neg log pval**	**number of genes**	**GeneIDs**
Cell adhesion	0.000120361	3.919514213	6	Nlgn1, Gpr98, Cpxm2, Cdhr3, Nell1, Nrp1
Cell junction	0.000136725	3.864152068	7	Nlgn1, Gphn, Anks1b, Unc13c, Dennd1a, Gabbr2, Shroom3
Synapse	0.000286469	3.542922368	6	Nlgn1, Gphn, Anks1b, Unc13c, Dennd1a, Gabbr2
Cell projection	0.00258205	2.588035352	5	Smo, Anks1b, Dennd1a, Gabbr2, Nrp1
Cell-cell adhesion	0.0077048	2.11323863	3	Nlgn1, Gpr98, Cdhr3
Protein binding	0.00280261	2.552437333	20	Magi2, Kctd16, Nlgn1, Gpr98, Gstcd, Gphn, Bmper, Ston2, Meis1, Myo3b, Dennd1a, Wdtc1, Nell1, Gabbr2, Nrp1
Postsynaptic membrane	0.000966876	3.01462922	4	Nlgn1, Gphn, Anks1b, Gabbr2
Cell communication	0.00914359	2.038883256	6	Smo, Nlgn1, Gpr98, Maoa, Unc13c, Bmper
Cell-cell signaling	0.00918952	2.036707173	4	Smo, Nlgn1, Maoa, Unc13c
Synaptic transmission	0.0351746	1.453770833	3	Nlgn1, Maoa, Unc13c
Signaling	0.0486346	1.313054652	10	Smo, Magi2, Nlgn1, Gpr98, Maoa, Olfr593, Unc13c, Bmper, Gabbr2, Nrp1
Regulation of developmental process	0.00149985	2.823952173	7	Smo, Nlgn1, Meis1, Nell1, Nrp1, Ccl11, Shroom3
Anatomical structure development	0.0077048	2.11323863	10	Smo, Nlgn1, Gpr98, Gphn, Bmper, Meis1, Nell1, Nrp1, Ccl11, Shroom3
Growth	0.0297471	1.526555367	4	Smo, Wdtc1, Nrp1, Ccl11
Vasculature development	0.0332004	1.478856684	4	Smo, Meis1, Nrp1, Ccl11
Angiogenesis	0.0494972	1.305419368	3	Meis1, Nrp1, Ccl11
**LD>HD**				
**Subcategory name**	**pval**	**neg log pval**	**number of genes**	**GeneIDs**
Inositol phosphate metabolism	0.0188722	1.72417747	3	Pip5k1a, Inpp5a, Pik3c2g
Phosphatidylinositol signaing	0.0245305	1.6102936	3	Pip5k1a, Inpp5a, Pik3c2g
Phosphatidylinositol phosphate kinase	0.0439244	1.357294162	2	Pip5k1a, Pik3c2g
Cell adhesion	0.05073	1.294735138	5	Ncam1, Ptprm, Lama2, Cntn5, Krla2, Col19a1, Ctnnd2
Biological adhesion	0.05073	1.294735138	7	Ptprm, Lama2, Negr1, Cntn5, Krla2, Col19a1, Ctnnd2

Supplementary Table 3. List of gene ontology (GO) analysis hits. All intragenic DMRs were submitted for GO analysis using Genetrail using parameters listed in Methods. Subcategories of gene families associated with gene that had increased methylation in high dose (HD>LD) or low dose (LD>HD) are listed and p-values (Pvals) for each are provided. LD=low dose, HD=high dose
